# p38 MAP Kinase Signaling in Microglia Plays a Sex-Specific Protective Role in CNS Autoimmunity and Regulates Microglial Transcriptional States

**DOI:** 10.3389/fimmu.2021.715311

**Published:** 2021-10-11

**Authors:** Mahalia M. McGill, Alyssa R. Richman, Joseph R. Boyd, Bristy Sabikunnahar, Karolyn G. Lahue, Theresa L. Montgomery, Sydney Caldwell, Stella Varnum, Seth Frietze, Dimitry N. Krementsov

**Affiliations:** Department of Biomedical and Health Sciences, University of Vermont, Burlington, VT, United States

**Keywords:** multiple sclerosis, EAE (experimental autoimmune encephalomyelitis), microglia, sex differences, MAPK, p38 alpha, scRNAseq

## Abstract

Multiple sclerosis (MS) is an autoimmune demyelinating disease of the central nervous system, representing the leading cause of non-traumatic neurologic disease in young adults. This disease is three times more common in women, yet more severe in men, but the mechanisms underlying these sex differences remain largely unknown. MS is initiated by autoreactive T helper cells, but CNS-resident and CNS-infiltrating myeloid cells are the key proximal effector cells regulating disease pathology. We have previously shown that genetic ablation of p38α MAP kinase broadly in the myeloid lineage is protective in the autoimmune model of MS, experimental autoimmune encephalomyelitis (EAE), but only in females, and not males. To precisely define the mechanisms responsible, we used multiple genetic approaches and bone marrow chimeras to ablate p38α in microglial cells, peripheral myeloid cells, or both. Deletion of p38α in both cell types recapitulated the previous sex difference, with reduced EAE severity in females. Unexpectedly, deletion of p38α in the periphery was protective in both sexes. In contrast, deletion of p38α in microglia exacerbated EAE in males only, revealing opposing roles of p38α in microglia *vs*. periphery. Bulk transcriptional profiling revealed that p38α regulated the expression of distinct gene modules in male *vs*. female microglia. Single-cell transcriptional analysis of WT and p38α-deficient microglia isolated from the inflamed CNS revealed a diversity of complex microglial states, connected by distinct convergent transcriptional trajectories. In males, microglial p38α deficiency resulted in enhanced transition from homeostatic to disease-associated microglial states, with the downregulation of regulatory genes such as *Atf3*, *Rgs1*, *Socs3*, and *Btg2*, and increased expression of inflammatory genes such as *Cd74*, *Trem2*, and MHC class I and II genes. In females, the effect of p38α deficiency was divergent, exhibiting a unique transcriptional profile that included an upregulation of tissue protective genes, and a small subset of inflammatory genes that were also upregulated in males. Taken together, these results reveal a p38α-dependent sex-specific molecular pathway in microglia that is protective in CNS autoimmunity in males, suggesting that autoimmunity in males and females is driven by distinct cellular and molecular pathways, thus suggesting design of future sex-specific therapeutic approaches.

## Introduction

Multiple sclerosis (MS) is a multifactorial inflammatory disease of the central nervous system (CNS) characterized by demyelination, gliosis, axonal loss, and progressive neurological dysfunction. The etiology of MS is not well-understood, but current evidence suggests that activation of myelin-reactive CD4 T cells triggers an inflammatory cascade in the CNS, recruiting other immune cells that mediate the subsequent tissue destruction and pathology (1, 2). Although a number of therapies for MS have been developed recently, their efficacy and success rate are heterogeneous (3).

The principal animal model of MS, experimental autoimmune encephalomyelitis (EAE), is an autoimmune disease induced in several animal species by active immunization with CNS homogenate or specific myelin proteins/peptides, or by adoptive transfer of CD4 T cells reactive to these antigens. As in MS, autoreactive CD4 T cells enter the CNS to initiate inflammation and pathology, leading to clinical signs. The EAE model has been instrumental in improving our understanding of MS pathogenesis and the development of therapies (4).

Genetic studies in MS and EAE support a prominent role for cell-mediated immune mechanisms in disease susceptibility (5–7). Both T helper (Th)1 and Th17 cells, producing interferon (IFN)-γ and interleukin (IL)-17, respectively, are currently thought to be involved in the pathogenesis of MS and EAE (8). While CD4 T cells initiate the inflammatory cascade in CNS, other immune and CNS resident cells, such as macrophages, B cells, CD8 T cells, microglia, and astrocytes, are thought to mediate the tissue destruction and pathology (9). Damage to the oligodendrocyte-myelin-axon unit leads to impaired neural signal propagation, which in turn causes the neurological disability associated with MS. Secretion of toxic and/or pro-inflammatory mediators, such as tumor necrosis factor α (TNFα) and reactive oxygen species, by activated immune cells and CNS resident cells mediates the killing of oligodendrocytes and neurons. Immunoglobulin complex deposition and phagocytosis of myelin also contribute to tissue damage. Thus, therapies targeted at inhibiting these “secondary effector” cells, particularly myeloid cells, are likely to have significant benefit in terms of halting the pathological processes in MS (10).

With regard to microglia, the enigmatic brain-resident macrophage-like cells, their role is less clear compared with infiltrating myeloid cells. While some studies have suggested that they play a pathogenic role through the production of proinflammatory mediators (11, 12), other more recent studies have suggested protective, anti-inflammatory, or tissue-reparative roles (13, 14) [reviewed in detail in (15, 16)]. This issue is further complicated by the recent findings that suggest that microglia comprise a heterogeneous population of cells with multiple distinct subsets that change dynamically during neuroinflammation (17–19).

The p38 MAP kinase (MAPK) pathway is activated by inflammatory insults (e.g., toll-like receptor (TLR) ligands, cytokines), and stress stimuli (e.g., UV radiation, osmotic stress, DNA damage), *via* the upstream kinases MKK3 and MKK6 that are in turn regulated by numerous MKK kinases (20). Four isoforms of p38 MAPK (p38α, p38β, p38γ and p38δ) have been identified, each encoded by a separate gene. The ubiquitously expressed p38α (*MAPK14/Mapk14*) is the best characterized isoform, which is thought to be responsible for the vast majority of the inflammatory functions of this Ser/Thr kinase family (21). Over-activation of this pathway in autoimmune and/or inflammatory diseases, such as rheumatoid arthritis (RA) and inflammatory bowel disease (IBD) was recognized early on, and pharmacologic inhibitors of p38 have progressed as far as Phase II clinical trials in these diseases, though with limited success so far (21). In contrast, the p38 MAPK pathway has received little attention in MS or its models until more recently. Studies using genetic or pharmacological targeting of this kinase have suggested that p38 MAPK signaling in myeloid cells, T cells, dendritic cells (DCs), and potentially astrocytes or microglia may influence EAE pathogenesis, but many of the underlying mechanisms remain unclear (22). Interestingly, high-dimensional profiling of brain-resident myeloid cells (likely microglia) during EAE progression recently identified a signaling signature that prominently included activation of MAPK-activated protein kinase 2 (MK2, a.k.a. MAPKAPK2) and cyclic-AMP-responsive-element-binding protein (CREB) (23), both classically indicative of p38α activation (21).

In our previous studies, we found that pharmacologic inhibition of p38 MAPK was protective in EAE, but only in female mice (24). Using cell type-specific genetic targeting, we recapitulated this sex difference in mice deficient in *Mapk14* (encoding p38α) in the myeloid lineage (utilizing LysM-Cre; p38αCKO*^LysM^*). No obvious differences were detected in peripheral priming of encephalitogenic CD4+ T cells or myeloid cell subset composition. However, in the inflamed CNS at peak of EAE, we detected diminished encephalitogenic T cell responses and decreased activation of myeloid cells, specifically in p38αCKO^LysM^ female, but not male mice. Taken together, these results suggested that p38α signaling in myeloid cells plays a female-specific pathogenic role in EAE.

The LysM-Cre system drives Cre activity in a variety of myeloid cells, primarily including macrophages, granulocytes, monocytes, and to some extent microglial cells (12, 25), hence it is unclear whether the EAE phenotype observed in p38αCKO*^LysM^* mice are driven by a loss of p38α signaling in microglia, CNS-infiltrating myeloid cells, or both. To address this question, we took advantage of the *Cx3cr1-Cre* (*constitutive*) and *Cx3cr1-CreER* (*tamoxifen-inducible*) systems recently developed by the Jung group (12, 26). Both systems take advantage of high and constitutive *Cx3cr1* expression in microglia, which is also expressed on some subsets of peripheral monocytes. The tamoxifen-inducible version also allows for more selective targeting of microglia, taking advantage of the short-lived nature of peripheral monocytes, and the long-lived, self-renewing nature of microglia. We utilized these approaches to delete p38α in microglia and/or peripheral myeloid cells in the EAE model (depicted in **Figure S1**).

Our results demonstrate that p38α signaling in peripheral cells plays a pro-inflammatory role in both males and females, while p38α signaling in microglia plays a protective role only in males. Single cell and bulk transcriptomics revealed that p38α signaling in male but not female microglia promotes the maintenance of homeostatic/anti-inflammatory gene expression programs, and delays the appearance of so-called disease-associated microglia. These results uncover novel molecular pathways underlying sex differences in the pathogenesis of CNS autoimmunity, and suggest that design of therapeutic strategies for autoimmune disease should take biological sex into consideration.

## Materials and Methods

### Animals and Genetic Models

C57BL/6 (B6) mice expressing a floxed allele of *Mapk14*/p38α, B6.129-Mapk14^tm1.2Otsu^ (p38α*^fl/fl^*) (27) were crossed to mice constitutively expressing Cre recombinase under the control of the endogenous *Cx3cr1* promoter (B6J.B6N(Cg)-Cx3cr1^tm1.1(cre)Jung^; Cx3cr1-Cre) or the tamoxifen-inducible Cre-ER fusion gene under the control of the endogenous *Cx3cr1* promoter (B6.129P2(C)-Cx3cr1^tm2.1(cre/ERT2)Jung^/J; Cx3cr1-CreER), originally generated by Jung and colleagues (26). Both transgene alleles disrupt the normal *Cx3cr1* allele and thus they were maintained and studied as heterozygous. B6.SJL-*Ptprc^a^Pepc^b^*/BoyCrCrl (B6.CD45.1) congenic mice were purchased from NCI/Charles River. B6.Cg-Gt(ROSA)26Sor^tm3(CAG-EYFP)Hze^/J (ROSA26-flox-STOP-EYFP) reporter mice (28) were obtained from Jackson Laboratories.

For inducible deletion of p38α microglia, 6-10 week old p38α*^fl/fl^* mice (littermates expressing or not the Cx3cr1-CreER transgene) were injected i.p. with 2.4 mg Tamoxifen (Sigma, USA) for 4 consecutive days. Tamoxifen was dissolved in 100% ethanol at 100 mg/ml, followed by 1:8.3 dilution in corn oil (Sigma, USA), administered in 200 μl total volume per mouse.

All experimental mice were bred and housed in a single room within the vivarium at the University of Vermont, with the exception of B6.CD45.1 mice, which were directly purchased from NCI/Charles River for experimentation. The experimental procedures used in this study were approved by the Animal Care and Use Committee of the University of Vermont.

### Radiation Bone Marrow Chimeras

Reciprocal bone marrow chimeras between B6.CD45.1 mice and p38α*^fl/fl^* mice (littermates expressing or not the Cx3cr1-Cre transgene) were generated as follows. 8-12 week old recipient mice were irradiated twice with 550 rads 4-6 hours apart, followed by i.v. administration of 10 million whole bone marrow cells from the respective unmanipulated 8-12 week old sex-matched donors. Lead shields were not used to cover the head or any part of the body of the mice during irradiation (we found that their use was unnecessary to prevent microglial replacement, and it impaired efficient bone marrow replacement). The resulting chimeras were rested for 8 weeks to allow for maximal reconstitution prior to induction of EAE or other experimentation.

### Induction and Evaluation of EAE

EAE was induced using the 2×MOG_35-55_/CFA protocol, as previously described (29). Mice were injected subcutaneously with 0.1 mL of emulsion containing 0.1 mg of myelin oligodendrocyte glycoprotein peptide 35-55 (MOG_35-55_) peptide (Anaspec Inc., MA, USA) in PBS and 50% complete Freund’s adjuvant (CFA; Sigma, USA) on day 0 on the lower flanks (50 µl per flank), followed by an identical injection on upper flanks on day 7. CFA was supplemented with 4 mg/mL *Mycobacterium tuberculosis* H37Ra (Difco, USA). Pertussis toxin (PTX) was not used in this induction protocol because the molecular and cellular targets and mechanism of PTX in EAE remain poorly defined, and because this protocol (unlike that with PTX) results in moderate, rather than maximal disease severity, allowing us to detect changes in severity in either direction (exacerbation or amelioration). Starting on day 10, mice were scored visually, as follows: 0.5 - partial loss of tail tone, 1 – full loss of tail tone, 2 - loss of tail tone and weakened hind limbs, 3 - hind limb paralysis, 4 - hind limb paralysis and incontinence, 5 - quadriplegia or death. EAE scoring was performed by an observer blinded to the animals’ genotypes. Significance of differences in overall disease course was determined using two-way ANOVA, as previously described (30), using the genotype*time interaction term to evaluate significance of differences between the groups.

We note that substantial differences in baseline EAE severity across the different genetic or bone marrow chimeric models were observed. This is likely due to several factors, as follows. First, these experiments were done across a span of 4 years, with each strain studied at different time. During this time, different batches of EAE induction reagents were used, and different investigators performed the experiments. Second, each strain used, including CD45.1 congenic mice in bone marrow chimera experiments, represents a unique genetic background, which in all cases is not pure B6/J (see above). Lastly, we note that the treatments to generate bone marrow chimera models (irradiation and reconstitution) and inducible deletion in microglia (tamoxifen treatment) can also impact EAE susceptibility. Importantly, in all cases, we used comparisons between CKO and “WT” transgene-negative littermate controls that were immunized simultaneously, treated identically, and generated from same parents, thus minimizing genetic differences and experiment-to-experiment variability.

### Cell Isolation and Flow Cytometry

Single cells from the spleen were isolated by homogenization and red blood cell lysis using ammonium chloride solution (StemCell, USA). Mononuclear cells from the CNS were isolated as follows. Mice were deeply anesthetized under isoflurane and transcardially perfused with ice cold PBS. Brain was dissected from the skull, and spinal cord was dissected from the spinal column and placed on ice, followed by Dounce homogenization in ice cold PBS. The cell suspension was washed and loaded onto a 37%/70% Percoll gradient, followed by centrifugation. Mononuclear cells were isolated from the Percoll interphase, washed, and processed for staining.

Cells were incubated with Live-Dead fixable stain (either UV-Blue or NearIR; Life Technologies, USA), followed by surface staining using fluorophore-conjugated antibodies (Biolegend, USA) against the following markers in various combinations: CD11b, CD11c, CD45, CD45.1, CD45.2, TCRβ, CD4, CD8, CD19, CX3CR1, Ly6C, and Ly6G, followed by fixation in 1% paraformaldehyde. Cells were processed using an LSRII flow cytometer (BD Biosciences, USA). Flow cytometry analysis was performed using FlowJo software v10 (BD Biosciences, USA).

### Microglial Cell Sorting and RNA Isolation

Cells were isolated from the spinal cord using Percoll gradient, as described above. Microglia were purified by fluorescence-activated cell sorting (FACS) using fluorophore-conjugated antibodies against cell surface markers as described in the Results section. For microarray analysis, cells were sorted into tubes containing RLT lysis buffer (Qiagen, USA), with the cell volume:RLT volume ratio not exceeding 1:4. RNA was isolated using the Qiagen RNeasy Micro kit (Qiagen, USA). RNA quality was assessed using the Agilent Bioanalyzer 2100, and samples were selected for downstream analysis based on RNA integrity number (typically 6-9). RNA quantity was determined using Qubit Fluorometric Quantification (Thermofisher, USA). For scRNAseq analysis, microglia were sorted into tubes without lysis buffer, spun down and resuspended in an appropriate volume, and loaded onto the 10x Chromium flow cell.

### Bulk Transcriptional Profiling by Microarray

Microarray analysis was performed at the UVM’s Vermont Integrative Genomics Resource (VIGR) facility using the Mouse Affymetrix Clariom S Genechip and the GeneChip™ WT Pico Target Preparation reagent kit (ThermoFisher 9026220) as described by the manufacturer’s procedures and previously published (31).

Raw intensity CEL files were imported into Expression Console software (Affymetrix, USA), and CHP files were generated for gene level analysis. CHP files were imported into Transcriptome Analysis Console (TAC) software v4.0.0.25 (Affymetrix, USA), and gene level differential expression analysis was performed using the default ANOVA settings (e-Bayesian method). All microarray data have been deposited into the Gene Expression Omnibus (GEO) database (accession number GSE180864).

### Pathway Analysis

Pathway analysis was performed using Ingenuity Pathway Analysis™ (IPA; Qiagen, Inc, USA) software. The gene expression datasets containing differentially expressed genes (DEGs) between WT and CKO microglia (cut-off filter of fold-change > 2 and ANOVA P< 0.05) were exported from TAC software and uploaded into IPA. The IPA Core Analysis function was applied to DEG sets. The Canonical Pathway function was used to identify the top canonical pathways (P < 0.01, Z score > |2|) affected by the DEGs. The sign and magnitude of the Z scores are indicative of the predicted strength and direction of the p38αCKO effect. The Upstream Regulator analysis function was similarly used to predict Z scores and P-values for putative upstream regulators.

### Single Cell RNA Sequencing and Analysis – Male Microglia

FACS-isolated spinal cord microglia were loaded onto the 10x Genomics Chromium flow cell (one biological replicate sample per well), followed by GEM formation and cell barcoding. Single cell cDNA libraries were constructed using the Chromium Single Cell 3’ v3 Kit (10x Genomics, USA). Libraries were constructed using 16 cycles of PCR amplification, per manufacturer’s recommendation. Libraries were combined using an equimolar pooling strategy and sequenced on a HiSeq 2500 platform (Illumina, USA). All raw scRNAseq data and metadata have been deposited in GEO, accession number GSE185045.

Reads were mapped to the mouse genome using the default settings in CellRanger software (10x Genomics). Initial QC was performed using the default CellRanger settings. Cells were sequenced at ~100,000 reads per cell, and ~2,500 genes per cell were detected. A gene count matrix table was generated and imported into Seurat v3 (32). Additional manual QC steps in Seurat v3 revealed a cell cluster containing cells containing a high percentage of mitochondrial transcripts and low gene count, this cluster was removed as presumptively dead/dying cells (33). The following numbers of cells were included in the final analysis: WT (n=3) – 455, 321, 117 cells; CKO (n=3) – 291, 570, 567 cells; for a total of 2,321 cells analyzed. For cell clustering and dimensionality reduction, UMAP was implemented in Seurat v3 using 7 principal components, with this number chosen based on manual inspection of elbow and jackstraw plots. UMAP was performed using anchored/integrated analysis of WT and CKO samples together.

Find cluster markers command in Seurat v3 was used to identify signature genes for each cell cluster, using the following criteria: genes expressed in >25% of the cells in a cluster, with a Log2(FoldChange)>0.25 and Padj<0.05 for differential expression compared with the other clusters (full gene list is provided in **Supplementary File 2**). Heatmaps of select cluster signature genes (**Table 1**) were generated using average expression for all cells in each cluster. The same signature genes were used as “cluster modules” to assess gene expression across multiple clusters.

**Table 1:** Signature marker genes of microglial states and other cell types identified by scRNAseq analysis in males.

Name	Description	Signature Genes
hMG1	classic homeostatic	*Fos/Jun, P2ry12, Cx3cr1, Rgs1/2, Atf3, Btg2, Dusp1*
hMG2	homeostatic immune surveillance	*Son, Tra2b, Fus, Malat1, Mef2a, Mef2c, mt-genes*
DAM1	disease associated; MHC I-high	*Cd9, Cd63, Lamp1, Apoe, B2m, MHC I genes*
DAM2	disease associated; MHC II-high	*MHCII genes, Cd74, Saa3, Ccl5, Cxcl9, Spp1, Apoe, Apoc1*
DAM3	transitional disease associated	*Ercc5, Naaa, Ly86, Mcm6, Ccl4*
pMG1	proliferative, inflammatory	*Topo2a, Mki67, Ube2c, Birc5, Hist1h2ap, Cxcl10*
pMG2	proliferative, interferon signature	*Stmn1, Ube2c, Birc5, Ifi27l2a, Isg15*
iMG	ion homeostasis, disease associated	*Ftl1, Fth1, Atox1, Rps26, Rplp1, Cd52, Tyrobp*
Neut	neutrophils	*Gda, S100a6, Ly6g, Ngp, S100a8*
T cells	T cells	*Trbc2, Trac, Cd3g, Cd3d, Lck*

### Single Cell RNA Sequencing and Analysis – Female Microglia

Single cell RNA sequencing and analysis of female microglia was performed essentially as described above for males, with modifications as follows. During fluorescent antibody labeling for FACS, each individual biological sample (n=3 total per genotype) was also labeled with a unique TotalSeq-B™ oligonucleotide barcoded antibody cocktail targeting pan-hematopoietic markers CD45 (clone 30-F11) and MHC class I (clone M1/42), hashtag numbers 0303-0308, as well as TotalSeq™-B 0238 Rat IgG2a isotype control (Biolegend, Inc, USA). After FACS, three individual biological samples were pooled by genotype, and loaded into a single well on a 10x Genomics Chromium flow cell, followed by gene expression library construction using the Chromium Single Cell 3’ v3 Kit (10x Genomics, USA), with the additional cell surface feature barcoding library construction implemented as recommended by manufacturer. The gene expression libraries and cell surface feature libraries were pooled at a ratio of 4:1 and sequenced as above.

Gene expression reads were mapped to the mouse genome as above and hashtag reads were quantified in each cell using CellRanger. QC filtering to ensure high quality single cells was performed by retaining cells with >250 and <2500 genes, and mitochondrial reads <20% of total reads. Hashtag counts were used to demultiplex individual samples and remove cell doublets using Seurat v3, as previously described (34). For clustering and DEG analysis, demultiplexed female data and male data were harmonized into a single Seurat object using 1671 cell anchors identified for dataset integration (32). Clustering analysis was performed with UMAP using 7 principal components. Genes for cluster identification were identified using genes with expression in >10% of the cells in a cluster, with a Log2(FoldChange)>0.25 and Padj<0.05 for differential expression compared with the other clusters. All differential gene expression between p38αCKO and WT microglia was determined using parameters P<0.05 and Log2(FoldChange)>|0.2|.

### Inference of Single Cell Transcriptional Trajectories

For inference of transcriptional trajectories, Monocle 3 (v0.2.2) (35) was applied to the subset of cells assigned to hMG1, hMG2, DAM1, DAM2, and DAM3 clusters. 5 principal components were used for preprocessing and a minimal branch length of 4 was used for learning the graph. Based on the resulting trajectory, subgraphs for a root and branch terminating at DAM1 was chosen as well as 4 distinct roots and branches terminating at DAM2. Gene expression in each subgraph was tested for spatial correlation using with Moran’s I. A gene was considered statistically significant if it met a minimum Moran’s correlation of 0.2 and a maximum q-value of 0.05 (full list is provided in **Supplementary File 3**). Significant genes were assigned to modules and modules were assessed for Gene Ontology: Biological Process enrichment using clusterProfiler(v3.14.3) (36). Seurat v3 was used to generate heatmaps of scaled expression data for the top 10 genes per module by Moran’s I statistic with cells sorted by pseudotime. The change in cell cluster composition with pseudotime was visualized by dividing the pseudotime span into 20 bins of equal cell count and viewing the fraction of each cell cluster per bin.

### Statistical Analyses

Statistical analyses not pertaining to transcriptomic data were carried out using GraphPad Prism software, version 8. Details of the analyses are provided in the figure legends and below. All statistical tests were two-sided, and adjustments for multiple comparisons were made as indicated, where appropriate. All center values represent the mean, and error bars represent the standard error of the mean. P-values below 0.05 were considered significant. Sample sizes for animal experiments were chosen based on previous experience with similar analyses. Co-housed littermate controls were used for all comparisons between genetically modified animals. Investigators were blinded to the genotypes. Analyses of EAE clinical scores were performed as described in the EAE section, above.

## Results

### Deletion of p38α in Both Microglia and Myeloid Lineages Using Constitutive *Cx3cr1-*Cre Is Protective in EAE in Females

**Figure 1:**
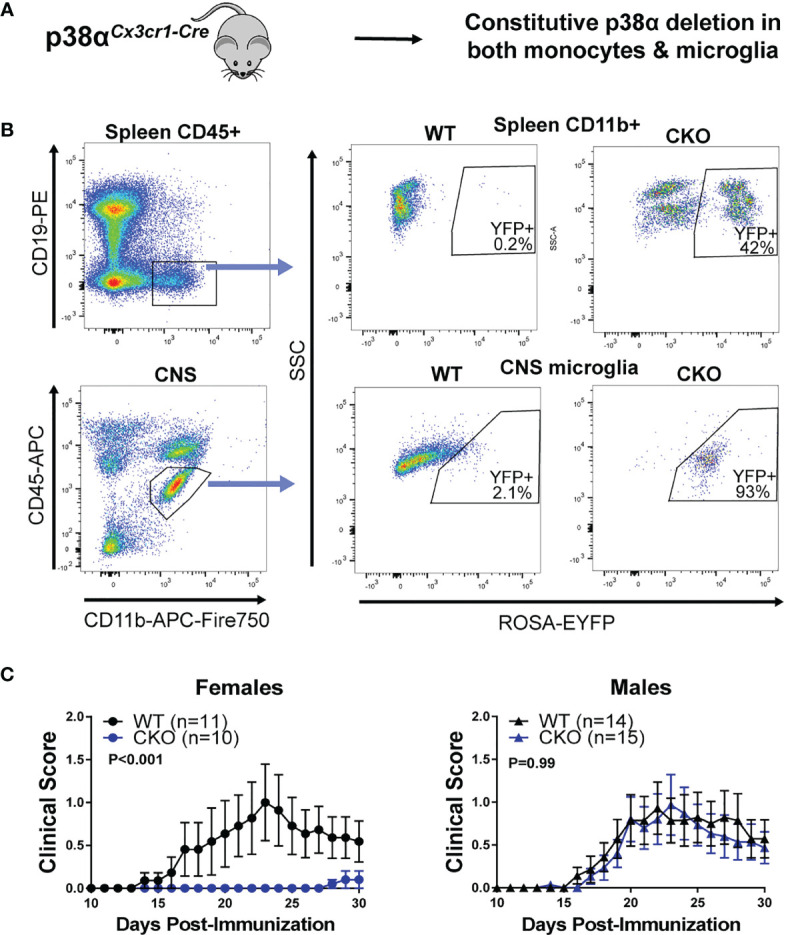
Combined peripheral myeloid and microglial deletion of p38α reduces EAE severity in only in female mice. **(A)** A schematic illustrating the approach to targeting of genetic deletion to specific cell types used in Figure 1. **(B)** Spleen cells and CNS cells were isolated from male naïve p38αCKO*^Cx3cr1^* ROSA-EYFP+ mice as described in the Materials and Methods and analyzed by flow cytometry. Top panel shows reporter EYFP expression in spleen myeloid cells (CD45^+^ CD19^-^ CD11b^+^) from CKO (p38αCKO*^Cx3cr1^* ROSA-EYFP+) or WT (p38α^fl/fl^ Cx3cr1-Cre-negative ROSA-EYFP+ littermates) mice. Bottom panel shows EYFP expression in CNS microglia (CD11b^+^ CD45^INT^). **(C)** EAE was induced and evaluated in female and male p38αCKO*^Cx3cr1^* and WT control mice (p38α^fl/fl^ Cx3cr1-Cre-negative littermates) as described in Materials and Methods. P value indicates significance of difference in overall EAE course between WT and CKO, calculated by two-way ANOVA, as detailed in Materials and Methods. Sample size for each sex/genotype is indicated in parentheses in the panel legends.

To determine the role of p38α in microglia and peripheral myeloid cells, we employed 4 complementary approaches (depicted in **Figure S1** and each respective figure). First, in order to corroborate our previous findings obtained using pan-myeloid-specific p38αCKO*^LysM^* mice, we crossed constitutive *Cx3cr1*-Cre mice to p38α^fl/fl^ mice to generate p38αCKO*^Cx3cr1^* mice, deficient in p38α in peripheral myeloid lineages and in microglia (**Figure 1A**). These mice were additionally crossed to the ROSA26-flox-STOP-EYFP reporter mice (28) to localize Cre activity. As expected, EYFP expression was observed in the majority of microglial cells, which constitutively express *Cx3cr1*, as well as in a large fraction of peripheral myeloid cells, many of which express *Cx3cr1* at some point in their development (**Figure 1B**). EAE was induced in male and female p38αCKO*^Cx3cr1^* (p38α^fl/fl^ and *Cx3cr1-Cre* hemizygous) and wild-type (WT) controls (p38α^fl/fl^ *Cx3cr1*-Cre-negative littermates), as described in Methods. Compared with WT, female p38αCKO*^Cx3cr1^* mice exhibited significantly reduced EAE severity (**Figure 1C**). However, no difference in EAE course was observed between male p38αCKO*^Cx3cr1^* and WT mice (**Figure 1C**). Taken together, these results demonstrate that deletion of p38α in microglia and myeloid lineages in female mice using *Cx3cr1-*Cre is protective in EAE. These results are in line with our published sex difference in pan-myeloid-specific p38αCKO*^LysM^* mice (24).

### Deletion of p38α in Bone-Marrow Derived Myeloid Cells Is Protective in Both Sexes

**Figure 2:**
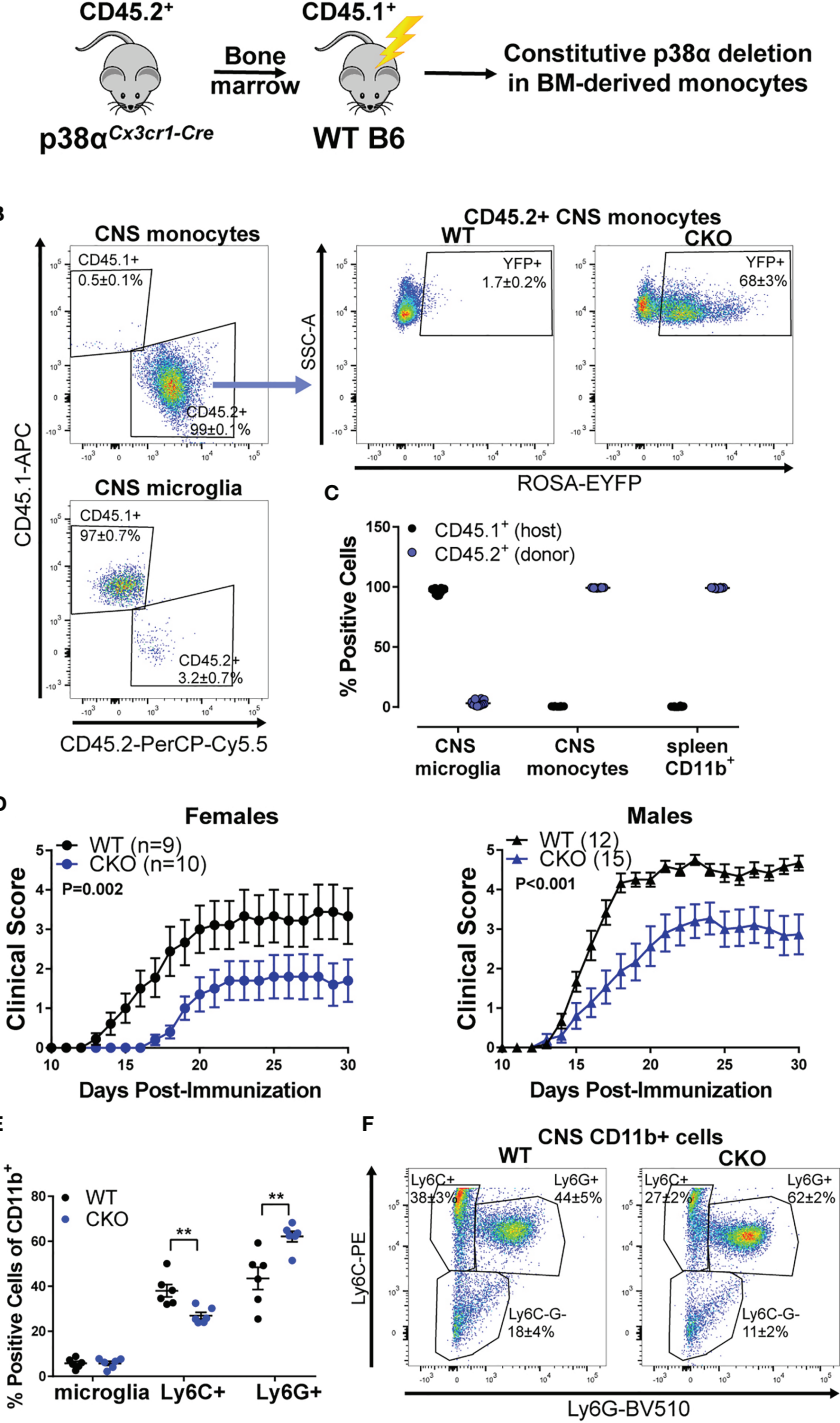
Deletion of p38α in BM-derived myeloid cells reduces EAE severity in female and male mice. Bone marrow chimeric mice were constructed by transplanting bone marrow from CD45.2^+^ donors (either p38αCKO*^Cx3cr1^* or WT littermate Cre-negative controls) to CD45.1^+^ WT recipients (see Materials and Methods), as illustrated in the schematic in panel **(A)**. **(B)** CNS cells were isolated from chimeric p38αCKO*^Cx3cr1^* BM→WT (CKO) and WT BM→WT (WT) male mice at day 30 post EAE induction and analyzed by flow cytometry. Left side - representative plots showing CD45.1 (host) and CD45.2 (donor) marker expression for CNS monocytes (CD45^+^ CD11b^+^ Ly6C^+^) and microglia (CD45^+^ CD11b^+^ Ly6C^-^ Ly6G^-^ MHCII^-^ CD11c^-^). Right side – representative plot showing EYFP reporter expression in CD45.2^+^ CNS monocytes (gated as above) in WT and CKO mice. Frequencies are shown as mean ± SEM. **(C)** Quantification of frequencies of CD45.1^+^ (host) and CD45.2^+^ (donor) cells in CNS monocytes and microglia [gated as in **(A)**] and spleen CD45^+^ CD11b^+^ myeloid cells. **(D)** EAE course in chimeric p38αCKO*^Cx3cr1^* BM→WT (CKO) and WT BM→WT (WT) male and female mice. P value indicates significance of difference in EAE course between WT and CKO, calculated as in . Sample size for each sex/genotype is indicated in parentheses in the panel legends. **(E, F)** Flow cytometric analysis of CNS-infiltrating immune cells isolated from male WT and CKO at day 30 post EAE induction. Frequencies of the following populations (within the parental CD11b^+^ population) were calculated: microglia (CD45^+^ CD11b^+^ Ly6C^-^ Ly6G^-^ CXCR1^+^ CD45.2^int^), Ly6C^+^ monocytes (CD45^+^ CD11b^+^ Ly6C^hi^ Ly6G^-^), and Ly6G+ granulocytes (CD45^+^ CD11b^+^ Ly6C^int^ Ly6G^+^). Summary data are shown in **(E)** and representative plots shown in **(F)**, with the numbers indicating frequency mean ± SEM. Significance of differences between WT and CKO were determined using t-test, and indicated as follows: **P < 0.01.

To tease apart the relative contribution of peripheral myeloid cells to the EAE phenotypes described above, we employed a bone marrow (BM) chimera approach, taking advantage of the relative resistance of microglia to irradiation (see **Figure 2A**). In order to track the host/donor origin in our cells of interest, irradiated C57BL/6 CD45.1 (B6.CD45.1) congenic mice (carrying the SJL/J-derived CD45.1 allele) were used as recipients of bone marrow transplants from either p38αCKO*^Cx3cr1^* or WT (Cre-negative p38α^f/f^ littermate) mice (which carry the B6 CD45.2 allele). In the resulting chimeric mice, even in the inflamed CNS during EAE, ~97% of the microglia were host-derived (CD45.1^+^) and hence WT, while ~99% of CNS-invading peripheral monocytes were donor-derived (CD45.2^+^) and hence either WT control (p38α^fl/fl^) or p38α-deficient, as indicated by EYFP reporter activity in ~70% of monocytes (**Figures 2B, C**). Similarly, in the spleen, ~99% of CD11b^+^ myeloid cells were donor-derived (**Figure 2C**). Thus, this bone marrow chimera approach allows for deletion of p38α primarily in peripheral myeloid cells and not in microglia.

EAE was induced in the resulting chimeric p38αCKO*^Cx3cr1^* BM→WT host and WT control BM→WT host, female and male mice. Compared with recipients of control WT bone marrow, mice receiving p38αCKO*^Cx3cr1^* bone marrow had significantly reduced EAE severity, independent of sex (**Figure 2D**). Profiling of CNS-infiltrating immune cells by flow cytometry revealed that, compared with WT, p38αCKO*^Cx3cr1^* bone marrow recipients had comparable proportions of microglia, but a decrease in the proportion of infiltrating Ly6C^+^ myeloid cells, and an increase in the proportion of Ly6G^+^ myeloid cells (**Figures 2E, F**), suggesting a reduction in pathogenic monocytes (37). Taken together, these results suggest that deletion of p38α in peripheral myeloid cells is protective in EAE in both females and males. This raises the question as to why deletion of p38α in these cells and microglia together is protective only in females (**Figure 1C**).

### Bone Marrow Chimeric WT→p38αCKO*^Cx3cr1^* Mice Show Poor Specificity of Conditional Gene Targeting in Microglia

**Figure 3:**
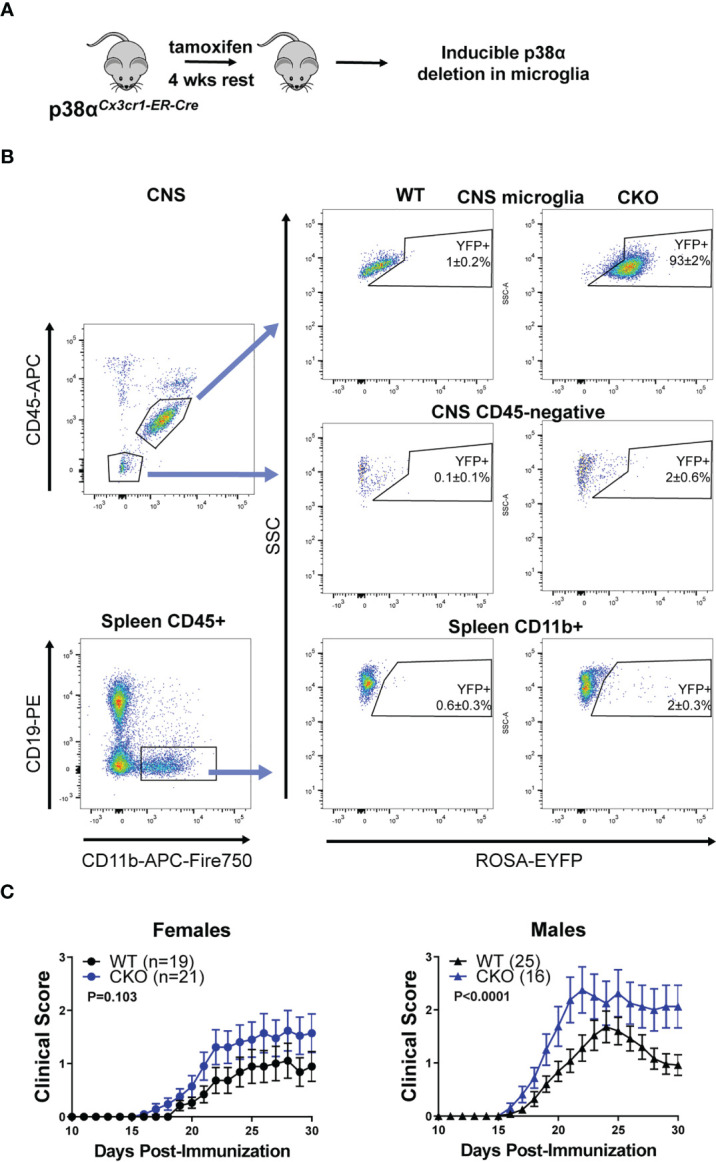
Deletion of p38α in microglia using inducible *Cx3cr1*-CreER exacerbates EAE in male mice. **(A)** A schematic illustrating the approach to targeting of genetic deletion to specific cell types used in Figure 3. **(B)** 6-12 week old ROSA-EYFP+p38αCKO*^Cx3cr1-ER^* and WT control mice were treated with 2.4 mg tamoxifen i.p. for 4 days, followed by a 4 week rest period. Spleen cells and CNS mononuclear cells were isolated from p38αCKO*^Cx3cr1-ER^* mice as described in the Materials and Methods and analyzed by flow cytometry. Reporter EYFP expression in CNS microglia (top panel; CD11b^+^ CD45^INT^), CD45-negative CNS cells (middle panel), and spleen myeloid cells (CD45^+^ CD19^-^ CD11b^+^) isolated from CKO (p38αCKO*^Cx3cr1-ER^* ROSA-EYFP+) or WT (p38α^fl/fl^ Cre-negative ROSA-EYFP+ littermates) mice. **(C)** 6-12 week old female and male p38αCKO*^Cx3cr1-ER^* and WT control mice were treated with 2.4 mg tamoxifen i.p. for 4 days, followed by a 4 week rest period, at which point EAE was induced and evaluated as described in Materials and Methods. P value indicates significance of difference in EAE course between WT and CKO, calculated as in . Sample size for each sex/genotype is indicated in parenthesis the panel legends.

To address relative contribution of microglial cells to the EAE phenotypes described above, we employed two parallel approaches: (1) bone marrow chimeras using p38αCKO*^Cx3cr1^* mice as recipients (**Figure S2A**), and (2) the inducible *Cx3cr1*-CreER system (**Figure 3A**), the former relying on the relative resistance of microglia to irradiation and replacement by bone marrow derived cells (11). For the former approach, (WT) B6.CD45.1 bone marrow was transplanted into irradiated p38αCKO*^Cx3cr1^* or WT (p38α*^fl/fl^* littermate) recipients, thus hypothetically restricting the deletion of p38α to microglia. Similar to the reciprocal bone marrow chimeras above, ~98% of peripheral myeloid cells were donor-derived, while ~90% of microglia were host-derived (**Figures S2B, C**). However, EYFP reporter activity was observed only in a portion (~42%) of host microglia (**Figure S2B**), suggesting inefficient maintenance of deletion in this model. Furthermore, a significant fraction (~20%) of non-immune CD45-negative CNS resident cells exhibited EYFP reporter activity (**Figure S2B**), suggesting that deletion of our gene or interest is not restricted to microglia and may occur to varying degrees in multiple other CNS cell types (likely other glia and/or neurons), as reported recently (38). EAE experiments in these chimeric mice yielded variable results, with no consistent net effect of (attempted) p38α deletion in males or females (**Figure S2D**). These results suggest that this WT BM→p38αCKO*^Cx3cr1^* host chimeric model is not adequate to address the role of p38α in microglia in EAE.

### Deletion of p38α in Microglia Using *Cx3cr1-*CreER Is Protective in Males

As the alternative approach, we utilized the tamoxifen-inducible *Cx3cr1*-CreER model to selectively delete p38α in microglia (**Figure 3A**). p38α^f/f^ mice were crossed to *Cx3cr1*-CreER and ROSA26-STOP-EYFP mice to generate p38αCKO*^Cx3cr1-ER^* mice. p38αCKO*^Cx3cr1-ER^* mice and WT controls (p38α^f/f^ Cre-negative littermates) were treated with tamoxifen to induce Cre activity, followed by a 4 week rest period to allow any Cre-expressing peripheral myeloid cells to turn over owing to their short half-life (12). At this time point, the majority (~93%) of microglia retained the EYFP reporter, whereas peripheral myeloid cells in the spleen were mostly YFP-negative (~98%) (**Figure 3B**). No reporter expression was observed in CD45-negative CNS cells (**Figure 3B**). EAE was induced in p38αCKO*^Cx3cr1-ER^* and WT control mice at 4 weeks post-tamoxifen treatment. In female mice, although a trend towards more severe EAE was detected in p38αCKO*^Cx3cr1-ER^* compared with WT mice, it did not reach significance, despite a large sample size (**Figure 3C**). In contrast, in males, p38αCKO*^Cx3cr1-ER^* mice demonstrated a more pronounced and significant exacerbation of EAE course (**Figure 3C**). These results demonstrate that p38α signaling in microglia plays a protective role in EAE in males, but not females. Together with the findings that p38α in peripheral myeloid cells plays a pathogenic role in both males and females (**Figure 2**), these data suggest that, in males, opposing effects of p38α in these two cell types may cancel each other out in p38αCKO*^Cx3cr1^* (**Figure 1**) or p38αCKO*^LysM^* (24) mice (see Discussion).

### Identification of p38α-Dependent Gene Signatures in Male and Female Microglia Using Bulk Transcriptomics

**Figure 4:**
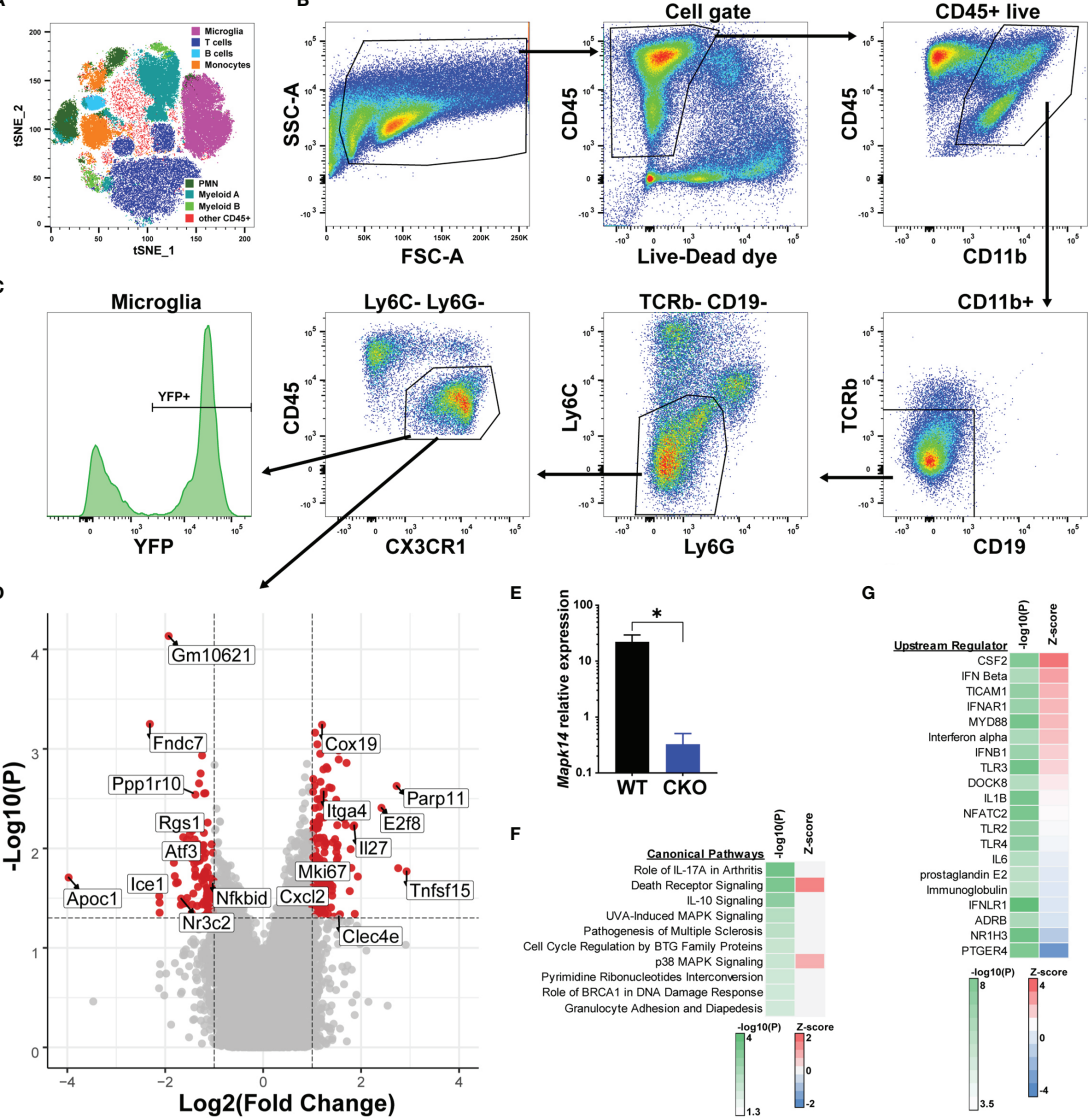
Bulk transcriptional profiling reveals p38α-dependent gene signatures in microglia. Male p38αCKO*^Cx3cr1-ER^* (CKO; n=4) and WT control (n=6) mice were treated with tamoxifen for 4 days, followed by a 4 week rest period, at which point EAE was induced. On day 21 post-EAE induction, mononuclear cells were isolated from the brain and spinal cord by Percoll gradient, followed by FACS analysis and isolation of microglia, followed by RNA isolation and microarray analysis (see Materials and Methods). **(A)** FlowJo-based tSNE analysis of FACS data demonstrating the major cell populations identified by FACS. **(B)** Representative gating scheme and FACS data for microglia isolation. **(C)** Reporter YFP expression in microglia. **(D)** Volcano plot demonstrating differential gene expression in CKO *vs*. WT microglia, passing the threshold of |FoldChange|>2 and P<0.05. Select genes are annotated. Positive fold change indicates higher expression in CKO. **(E)** qRT-PCR analysis of *Mapk14* (p38α) mRNA expression in sorted WT and CKO microglia, normalized by *B2m* house-keeping gene expression (*10000). **(F, G)** Genes differentially expressed in CKO *vs*. WT were subjected to bioinformatic analysis using Ingenuity software (see Materials and Methods). Top 10 canonical pathways, as identified by P-value of enrichment are shown in **(F)**, with Z-score indicating enhanced predicted activity in CKO. Top 20 predicted upstream regulator nodes, as identified by absolute Z score are shown in **(G)**.

In order to identify the molecular mechanisms underlying the exacerbated EAE in male p38αCKO*^Cx3cr1-ER^* mice, which lack p38α signaling selectively in microglia, we performed transcriptional profiling of microglia during acute EAE. Male p38αCKO*^Cx3cr1-ER^* and WT control littermates (p38α^f/f^ Cre-) were treated with tamoxifen to induce Cre activity, followed by EAE induction at 4 weeks post tamoxifen treatment, as above. On day 20 post-EAE induction, microglia were isolated from the brain and spinal cord using multi-parameter fluorescence-activated cell sorting (FACS), which could reliably distinguish resident microglia from the major populations of cells infiltrating the CNS (**Figure 4A**). Microglia were identified as CD11b^+^ CD45^int/lo^ CX3CR1^+^ Ly6C^-^ Ly6G^-^ TCRb^-^ CD19^-^ cells (**Figure 4B**), of which approximately 70% retained YFP reporter expression (**Figure 4C**). RNA was isolated from bulk FACS-isolated microglia, followed by transcriptional profiling by microarray (see Methods), using biological replicates (WT, n=6; CKO, n=4). A comparison between WT and p38α-deficient male microglia revealed 254 differentially expressed genes (DEGs), with 154 genes significantly upregulated in CKO relative to WT, and 100 genes significantly downregulated (fold change > 2, P<0.05) (**Figure 4D** and **Supplementary File 1**). The expression of *Mapk14* (encoding p38α) in CKO microglia was confirmed to be downregulated (by ~98%) by qRT-PCR (**Figure 4E**). Among the genes downregulated in CKO microglia, we noted several genes of interest with potential homeostatic, anti-inflammatory, regulatory, and/or anti-proliferative roles, including *Atf3*, *Rgs1*, *Btg2*, *Ppp1r10*, and *Nfkbid*. Among upregulated genes in CKO, we noted several pro-inflammatory genes, including *Cxcl2*, *Cxcl10*, *Tnfsf15*, and *Clec4e*, along with proliferation marker *Mki67*, although anti-inflammatory genes *Il27* and *Il1rn* were also upregulated. Altogether, these results suggest that exacerbated clinical EAE in male p38αCKO*^Cx3cr1-ER^* mice is driven by downregulation of immune regulatory and anti-proliferative genes, with upregulation of pro-inflammatory and proliferative genes.

Pathway analysis of p38α-dependent DEGs in males (see Methods) revealed enrichment of DEGs within autoimmune disease-associated pathways such as rheumatoid arthritis and MS, IL-10 and death receptor signaling, and as expected, p38 MAPK-related pathways (**Figure 4F**). An additional pathway of interest was Cell Cycle Regulation by BTG family proteins, including the DEGs *Btg2* and two transcriptional repressors, *E2f6* and *E2f8*. Despite robust enrichment of specific pathways, there was a lack of clear directionality in terms of effect of p38α deficiency, as inferred by Z-score analysis (**Figure 4F**). Upstream regulator analysis (see Methods) suggested that p38α-deficiency resulted in enhanced activity downstream of proinflammatory regulators such as CSF2 (encoding GM-CSF) and MYD88, as well as several members of the type I IFN pathway, while diminished signaling was predicted downstream of anti-inflammatory regulators such as ADRB and NR1H3 (39, 40), as well as prostaglandin signaling (PGE2 and PTGER4), which plays both pro- and anti-inflammatory roles in CNS autoimmunity (41) (**Figure 4G**).

Because p38α deletion in microglia significantly exacerbated EAE in males, but not in females (**Figure 3**), we next compared the impact of p38α deficiency in male microglia to that in females. Female p38αCKO*^Cx3cr1-ER^* and WT control littermates (p38α^f/f^ Cre-) were treated with tamoxifen to induce Cre activity, followed by EAE induction at 4 weeks post tamoxifen treatment, as above. On day 20 post EAE induction, microglia were isolated by FACS, followed by transcriptional profiling by microarray using biological replicates (WT, n=5; CKO, n=5). A comparison between WT and p38α-deficient female microglia identified 92 DEGs, with 34 genes significantly upregulated in CKO relative to WT, and 58 genes significantly downregulated (**Figure S3A** and **Supplementary File 1**). Next, we compared p38α-regulated DEGs in female *vs*. male microglia, which revealed several key patterns. First, p38α deletion in males and females resulted in a comparable number of downregulated genes (100 genes in males *vs* 58 in females), with significant overlap (13 genes, including *Ppp1r10, Adamts1, Hmox1* and 3 histone cluster genes). Interestingly, several pro-inflammatory genes were downregulated specifically in female p38α-deficient microglia, including *Il1b*, *Ccl3*, *Ccl4*, and *Ccr1*. In contrast, compared with males, p38α deletion in females resulted in a modest number of upregulated genes (34 genes in females *vs*. 154 in males), with zero overlap. Pathway analysis of sex-specific DEGs confirmed that p38α deficiency had a profoundly different impact on male *vs* female microglia, with pronounced upregulation of proinflammatory pathways in p38α-deficient males that was either absent or completely reversed in females (**Figures S3B, C**). Taken together, these results suggest that p38α regulates the expression of different transcriptional modules in males *vs*. females, and in particular negatively regulating a large number of proinflammatory pathways in males, but not females.

Of note, several of the above p38α-dependent DEGs identified in males have also been recently identified using single cell RNA sequencing (scRNAseq) as markers of various microglial transcriptional states/identities, including *Atf3* and *Btg2* during microglia differentiation and aging (42) and glioblastoma development (43); *Btg2*, *Cxcl2*, and *Cxcl10* during demyelination or CNS autoimmunity (42, 44, 45), and *Clec4a* during CNS inflammation (46). These data suggest that p38α-dependent DEGs identified by our bulk transcriptomic analysis may be a result of differential composition of heterogeneous subsets of microglia present in WT *vs* CKO mice. Alternatively, these DEGs could result from differential gene expression within specific subsets of microglia. Single cell transcriptomics could resolve these two alternative hypotheses.

### Single Cell Transcriptomic Analysis Reveals Distinct Transcriptional States and Trajectories of Microglia During Autoimmune Neuroinflammation

**Figure 5:**
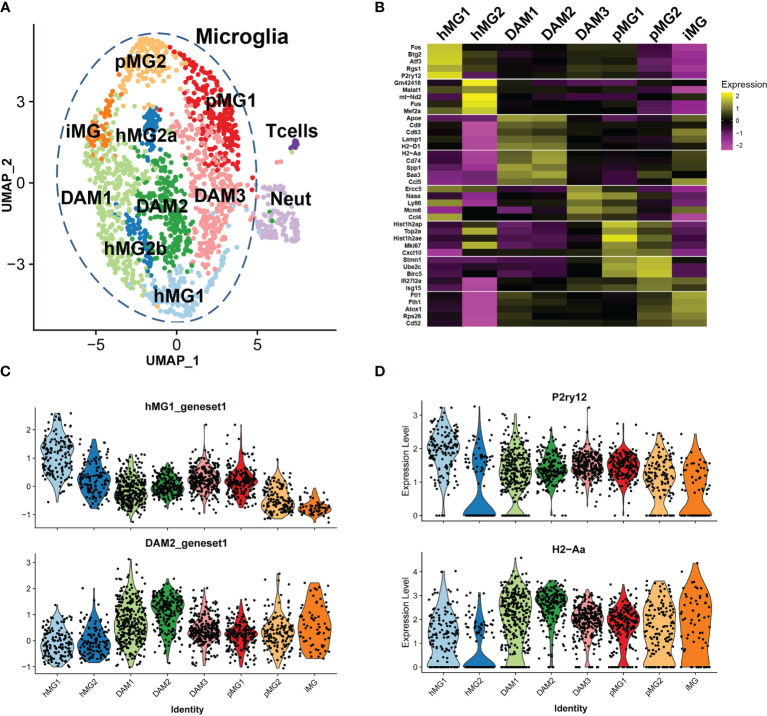
Single cell transcriptional analysis reveals heterogeneous transcriptional states of microglia in EAE. Microglia were isolated by FACS from male p38αCKO*^Cx3cr1-ER^* (CKO) and WT control mice (n=3 each) on day 21 post-EAE induction, as described in . ScRNAseq analysis was performed as described in Materials and Methods, using Seurat3 software. Combined analysis of WT and CKO cells is shown. **(A)** UMAP-based two-dimensional projection of cell clustering of major cell populations and transcriptional states. **(B)** Heatmap of signature gene markers for 8 microglial cell clusters. Row normalized gene expression is shown for 5 select significant cluster marker genes for each cell cluster. Average gene expression of all cells in each cluster is shown. **(C)** Violin plots demonstrating the expression of cluster module genes (as defined in **B**) for hMG1 (top) and DAM2 (bottom) modules, across 8 microglial clusters. **(D)** Violin plots demonstrating expression of select genes, *P2ry12* (top) and *H2-Aa* (bottom), across 8 microglial clusters.

Our bulk gene expression analysis suggested the existence of heterogeneous microglial states/subsets, some of which may be selectively impacted by p38α deficiency. In order to address microglial heterogeneity at a single cell level, we performed scRNAseq analysis of microglial cells isolated from the inflamed CNS of male p38αCKO*^Cx3cr1-ER^* mice and WT controls (p38α^f/f^ Cre- littermates) at peak EAE using FACS. We initially focused this analysis on males, because microglial p38α deficiency had the most profound impact on clinical EAE severity in this sex (**Figure 3**). Following cell sorting and isolation (as described above), single cell RNA sequencing (scRNAseq) was performed using the 10x Genomics Chromium platform and standard protocols (see Materials and Methods). Read processing, alignment and initial QC was performed using the CellRanger pipeline (10x Genomics), followed by final analysis using the Seurat v3 package (32). WT and p38αCKO*^Cx3cr1-ER^* biological replicates (n=3 per each genotype) were processed together using an anchored UMAP-based dimensionality reduction and single cell clustering analysis (see *Materials and Methods*), which revealed the presence of 10 clusters (**Figure 5A**).

Marker gene analysis was used to identify signature genes whose expression was elevated in the clusters of interest (see *Methods* and **Supplementary File 2**), and biological identities were inferred by comparison with previously published scRNAseq analyses of microglia and other cell types. Two clusters of cells expressed markers consistent with T cell (*e.g., Cd3g*, *Trbc2, Lck*) and neutrophil identity (*e.g., Ngp*, *S100a8*, *Ly6g*) (**Figures S4A, B** and **Table 1**). These clusters contained a low number of cells, and they likely represent contaminating non-microglial cells captured by the FACS procedure, serving as a convenient point of reference to compare distinct cell *types vs*. cell *states* in our analysis. The T cell and neutrophil clusters were discrete from the major cell supra-cluster comprised of microglia, with the latter identified by the expression of microglial signature genes *P2ry12, Tmem119*, and *Cx3cr1*, although the expression of these markers was heterogeneous (**Figure S4C** and **Table 1**), as expected under neuroinflammatory conditions (19).

The microglial cell supra-cluster was comprised of 8 smaller clusters, each representing distinct microglial states. A canonical homeostatic microglial cluster (designated **hMG1**) was identified, expressing high levels of homeostatic microglia markers *P2ry12*, *Cx3cr1*, and *Tmem119*, as well as *Rgs2*, *Atf3*, *Btg2*, and several members of the *Fos/Jun* family (**Figures 5B-D** and **Table 1**) (19). A second putative homeostatic microglial cell cluster (**hMG2**) expressed high levels of the transcription factors *Mef2a* and *Mef2c*, associated with homeostatic immune surveillance by microglia (47), and several genes involved in RNA processing, including *Son*, *Tra2b*, *Fus*, and *Malat1* (**Figure 5B**, **Figures S4D-E** and **Table 1**). This cluster also had elevated expression of several mitochondrial transcripts, suggesting that these cells may be prone to cell death (33), either during the isolation procedure or in the inflamed environment of the CNS during EAE. Two additional clusters (designated **DAM1** and **DAM2**) expressed signature genes consistent with so-called damage-associated microglia (DAM) transcriptional state previously described in models of Alzheimer’s disease and MS (18, 45, 48), including *Apoe*, *Trem2*, *Cd9*, *Ctsd*, and *Ctsb* (**Figure 5B**, **Table 1**). DAM1 expressed higher levels of MHC class I genes and phagocytosis-associated genes, such as *H2-D1*, *Cd9*, *Cd63*, *Lamp1*, whereas DAM2 expressed higher levels of genes associated with class II antigen presentation and inflammation, such as *Cd74*, *H2-Aa*, *Ccl5*, *Cxcl9*, *Saa3*, and *Nfkbia* (**Figure 5B** and **Table 1**). An additional cluster of microglia was found (designated **DAM3**), which clustered closely with DAM2, hMG1, and pMG1, characterized by upregulation of *Ccl4, Naaa*, *Mdm6*, *Ly86*, and *Ercc5* (**Table 1**). *Ccl4* expression in microglia has been associated with states appearing during aging and demyelination (42, 45), and *Naaa* with *Ly86* expression marked a distinct but related DAM population (45), hence this population could represent a transitional homeostatic/DAM state. We identified two proliferative cell clusters (designated as **pMG1** and **pMG2**), expressing proliferation marker genes *Topo2a*, *Mki67*, *Ube2c*, and *Birc5* (**Figure 5B** and **Table 1**), mirroring the signature of proliferative microglia predominant in the developing brain (42) and consistent with upregulation of proliferative markers in various DAM states during neuroinflammation (45). pMG1 also expressed *Cxcl10*, consistent with a DAM-like state (45), while pMG2 expressed interferon-induced genes *Ifi27l2a* and *Isg15*, consistent with the type I interferon signature observed during demyelination (42, 44) and proliferative repopulation following demyelination (49). A unique minor cluster of microglia (designated **iMG**) exhibited high expression of genes related to metal homeostasis (*Ftl1*, *Fth1*, and *Atox1*), and a large number of ribosomal component genes (**Table 1**). This signature is reminiscent of an embryonic-like “primitive” microglial state identified as a minor population existing in the developing post-natal brain (50), but may also may a reflect response to dysregulated CNS iron homeostasis, a hallmark of EAE and MS lesions (51). In line with this notion, iMG also expressed high levels of *Cd52* and *Tyrobp*, associated with DAM-like states (44, 48), suggesting that these cells are also activated by the neuroinflammatory state in EAE. Cell cycle analysis confirmed our identification of proliferative clusters of microglia (pMG1 and pMG2), which exhibited predominantly a G2/M signature, while DAM1, DAM2, and hMG1 were predominantly G1, and hMG2 were split ~50/50 between these two states (**Figure S4F**). Taken together, our scRNAseq analysis reveals the co-existence of diverse transcriptional states in microglia during ongoing autoimmune inflammation in the CNS.

### Transcriptional Trajectories of Microglia in EAE Reveal Distinct Homeostatic Inputs That Converge Upon Disease-Associated States

**Figure 6:**
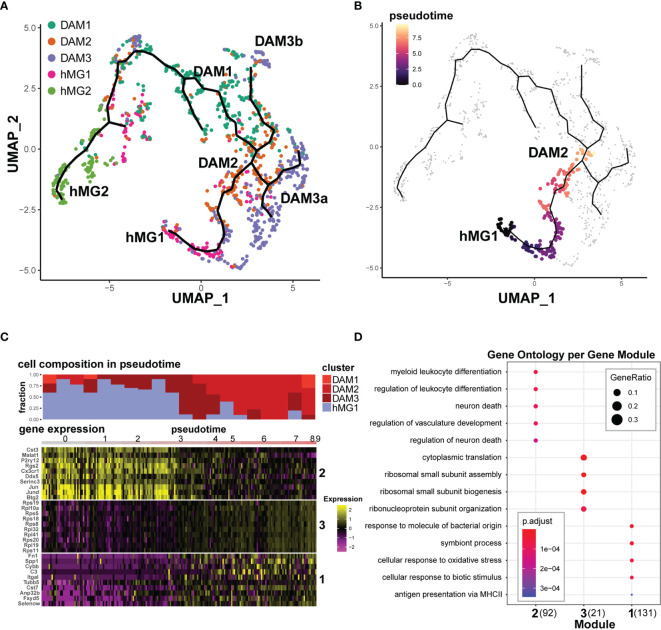
Transcriptional trajectories of microglia in EAE reveal distinct homeostatic inputs that converge upon disease-associated states. Select Seurat v3-defined microglial cell clusters (hMG1, hMG2, DAM1,2,3) were analyzed using Monocle 3, as described in the Materials and Methods. **(A)** Monocle 3-inferred transcriptional trajectories. **(B)** Trajectory between hMG1 and DAM2, manually selected for pseudotime analysis in **(C, D)**. **(C)** Top - cell composition over pseudotime along the hMG1 > DAM2 trajectory. Bottom – gene expression modules significantly associated with pseudotime. Heatmap shows gene expression for top 10 genes (ranked by Moran’s I statistic) for each module. **(D)** GO analysis of modules identified in **(C)**. The number in parentheses indicates the number of genes in each module.

Transcriptional states associated with cell states are not discrete, but exist as a continuum. In order to infer in an unbiased manner the transcriptional trajectories of microglial fates in the inflamed CNS, we employed a pseudotime trajectory analysis using the Monocle 3 package (35), focusing on the 5 most relevant and abundant Seurat v3-defined clusters: hMG1, hMG2, DAM1, DAM2, and DAM3. Monocle 3-inferred trajectories revealed a single interconnected partition comprised of all 5 clusters. This partition contained several branches that likely represent distinct putative homeostatic states/inputs (hMG1, hMG2, and possibly DAM3) that appeared to converge on DAM1 and DAM2 states in the center (**Figure 6A**). Interestingly, the hMG2 cluster appeared to primarily transition to DAM1, while hMG2 and DAM3 converged on DAM2, suggesting that the two predominant DAM states (DAM1 and DAM2) may arise from two distinct homeostatic lineages.

In order to reveal transcriptional signatures associated with differentiation between these states, we identified gene modules whose expression was significantly associated with defined branches in the pseudotime trajectories, using the Moran’s test statistics in Monocle 3. Focusing on the hMG1 > DAM2 differentiation branch (**Figure 6B**) demonstrated the presence of DAM3 microglia as an intermediate state (**Figures 6A, C** **)**, and revealed three gene modules exhibiting distinct kinetics (**Figure 6C**). One of these gene modules (Module 2) exhibited downregulation during this transition, including homeostatic genes *P2ry12, Cx3cr1, Rgs2, Btg2*, and *Fos/Jun* family genes (**Figure 6C** and **Supplementary File 3A**). Gene Ontology (GO) analysis of this gene module implicated myeloid cell differentiation and regulation of neuronal death (**Figure 6D**). The other two gene modules exhibited the opposite trajectory, with upregulation during the transition. Module 3 was comprised almost entirely of ribosomal subunit genes, while Module 1 was comprised not only of DAM-associated genes such as *Spp1*, *C3*, and *Cst7*, but also additional genes such as *Fn1* (encoding fibronectin), *Fxyd5* (encoding an Na,K-ATPase), *Selenow* (encoding a selenocysteine protein), and others (**Figure 6C**, **Supplementary File 3A**). Consistent with this, GO terms associated with this module included response to bacterial molecules, MHC class II antigen presentation, and response to oxidative stress (**Figure 6D**).

Focusing on the other major lineage trajectory branch, hMG2 > DAM1 (**Figure S5A**) similarly revealed three distinct gene modules with distinct kinetics (**Figure S5B**). Here, two modules exhibited progressive downregulation. One module (hMG2 > DAM1; Module 3) comprised of a number of mitochondrial genes and RNA processing-related genes *Malat1*, *Son*, *Fus*, etc, while the other (Module 2) was comprised predominantly of genes associated with proliferation, including *Mki67* and *Top2a* (**Figure S5B** and **Supplementary File 3B**), as also confirmed by GO analysis (**Figure S5C**). The third module (Module 1) was a very large heterogeneous module of 660 genes, which exhibited progressive upregulation during transition from hMG2 to DAM1 (**Figure S5B**). These genes included a number of ribosomal subunit genes, but also a large number of additional genes, including DAM-associated genes such as *Apoe, Cd52*, and *Lgals9*, MHC class I and class II genes, six different genes encoding selenocysteine family proteins, and others (**Figure S5B** and **Supplementary File 3B**), associated with heterogeneous GO term enrichment, including translation and nucleotide metabolism (**Figure S5C**).

Lastly, we analyzed two additional branches from DAM3a >DAM2 and DAM3b > DAM2 (**Figures S5D, E**). Each branch revealed a single relatively heterogeneous gene module, predominantly exhibiting progressive upregulation of expression during transition to DAM2 from DAM3a/b, including DAM-associated *Apoe, C1qa, Ctsb*, and *Lamp1*, along with prominent upregulation of MHC class I molecules, *H2-D1* and *H2-K1* (**Figures S5F, G** and **Supplementary File 3C, D**). GO analysis revealed that the DAM3a > DAM2 module was enriched for genes related to microglial activation and antigen presentation, while the DAM3b > DAM2 module was enriched for genes related to ribosome function and leukocyte cytotoxicity (**Figures S5H, I**). Taken together, these results map the continuum between microglial transcriptional states in the inflamed CNS, and suggest that different subsets of transcriptionally convergent disease-associated microglia arise *via* distinct lineages. Our results reveal a complex transcriptional adaptation in response to tissue damage that involves not only canonical inflammatory/immunological functions, but upregulation of translation machinery, ECM remodeling, ion homeostasis, alteration of RNA processing, and mitigation of oxidative stress.

### Single Cell Transcriptomics Reveals That p38α Signaling Regulates Microglial Subset-Specific Gene Expression Programs and Represses the Transition From Homeostatic to Inflammatory Microglia States in a Sex-Specific Manner

**Figure 7:**
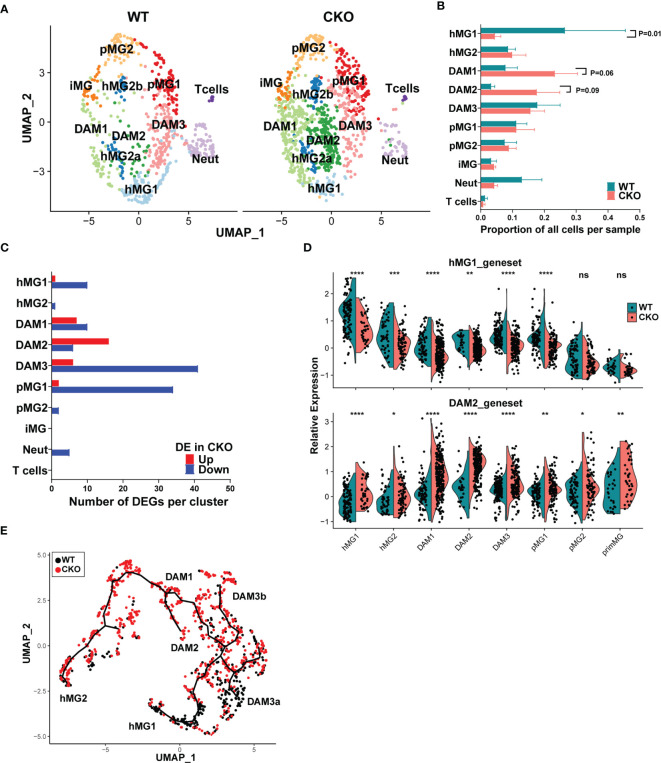
p38α in male microglia regulates subset-specific gene expression programs and represses the transition from homeostatic to inflammatory microglia states. Microglia were isolated from male p38αCKO*^Cx3cr1-ER^* (CKO; n=3) and WT control mice (n=3) during peak EAE and analyzed by scRNAseq as in **Figures 5**, **6**. **(A)** UMAP-based two-dimensional projection of cell clustering of major cell populations and transcriptional states between WT (right) and CKO (left) microglia. **(B)** Relative proportion of each cell cluster was determined by dividing the number of cells in a given cluster by the total number of cells in a biological replicate. **(C)** Distribution of genes differentially expressed between WT and CKO across the different cell clusters. Directionality of change in CKO relative to WT is shown. **(D)** Relative differential expression of the hMG1 (top) and DAM2 (bottom) gene modules across 8 microglial cell clusters. Significance of differences between WT and CKO are indicated as follows: *P < 0.05; **P < 0.01; ***P < 0.001; ****P < 0.0001; ns, not significant (P > 0.05). **(E)** Monocle 3-inferred trajectories between different microglial clusters were determined as in , highlighting the differential distribution of WT and CKO cells.

In order to understand how p38α deficiency impacted microglial states in males, we first examined the relative distribution of WT *vs* p38αCKO cells across the identified microglia clusters, using biological replicates to assess the level of variability between individual mice. Compared with WT cells, there was a significant reduction in the proportion of p38αCKO cells in the hMG1 cluster, with a trend toward a corresponding increase in DAM1 and DAM2 clusters (**Figures 7A, B**). To examine how p38α deficiency altered the transcriptome of individual clusters, we performed differential expression analysis comparing WT and CKO cells within each cluster. Across all clusters, total of 69 unique genes passed the differential expression threshold of P_adj_< 0.05. The distribution and directionality of DEGs across clusters was cluster-specific, with the most DEGs found in hMG1, DAM1/2/3, and pMG1 clusters, with minimal to no differential expression found in other clusters (**Figure 7C**). Many of the DEGs represented cluster signature/marker genes identified in the combined analysis above (**Figure 5B**), with marked downregulation of hMG1 and DAM3 markers, and upregulation of DAM1/2 markers (**Figure S6A**) in p38αCKO cells compared with WT. Interestingly, such downregulation of hMG1 and DAM3 marker genes was not restricted to homeostatic clusters, but was in fact pervasive across most cell clusters, while the converse was true for DAM1/2 markers (**Figure 7D** and **Figures S6B-D**). Expression of marker genes for the other clusters (hMG2, pMG1, pMG2 was mostly comparable between WT and p38αCKO cells (**Figures S6E-G**). Monocle 3 based comparison of cell state trajectories of WT and p38αCKO cells revealed a preferential increase in the DAM1/2 cells at the expense of hMG1 cells in p38αCKO, with an increased accumulation of the DAM3a transitional state in WT (**Figure 7E**). Taken together, these results suggest that the loss of p38α accelerates the transition from classically homeostatic microglial states to canonical DAM states, through early loss of homeostatic gene expression and imprinting of DAM signature genes.

A comparison of DEGs across the four clusters that exhibited the most DEGs (DAM1-3 and pMG1) revealed a small core group of genes dependent on p38α, whose expression was diminished in p38αCKO across at least 3 of these clusters, including *Btg2*, *Rgs1*, *Ccl4*, *Jun*, *Neat1*, *Rhob*, *Cx3cr1*, and 4 additional non-annotated genes, with most of these genes associated with homeostatic microglial function (**Figure S6H**). 2 genes were upregulated in p38αCKO cells across at least 3 clusters, *Lpl* and *Cd74*, both associated with DAM phenotypes, with several other DAM-associated genes upregulated in either or both of the DAM1/2 clusters, including *Apoe*, *Spp1, Trem2*, *Ccl5*, and a large number of ribosomal genes (**Figure S6H**). Interestingly, the DAM3 transitional cluster had the highest number of DEGs, with a predominant downregulation of genes, including the homeostatic/immune-regulatory genes *Atf3*, *Ppp1r10*, *Socs3*, *Nfkbiz, Tgfbi, Tgif*, and *Zfp36* (**Figure S6H**). Of note, several of the downregulated DEGs identified by scRNAseq analysis were also identified by bulk transcriptomics, including *Btg2*, *Atf3*, *Rgs1*, *Ppp1r10*, and *Adamts1*, representing p38α-regulated genes in multiple clusters of microglia. Other bulk DEGs, including *Cxcl2* and *Cxcl10*, which were upregulated, are markers of DAM (45), and their upregulation likely results from an increased abundance of DAM1/2 subsets in p38αCKO mice (**Figures 7A, B**). Pathway analysis of DEGs from the four cell clusters (DAM1-3 and pMG1) revealed an activation of pro-inflammatory signaling pathways, with a downregulation of anti-inflammatory pathways (**Figure S7A**). Upstream regulator analysis identified p38 MAPK as a predicted downregulated node, as expected (**Figures S7B, C**). Another highly downregulated node was TGFB1 (**Figures S7B, D**), consistent with role of TGFβ in microglial homeostasis. Taken together, our results demonstrate that p38α signaling in microglia in male mice promotes the maintenance of a homeostatic and regulatory gene expression program, and the loss of p38α promotes their transition to a DAM-like state, in association with exacerbated clinical disease.

**Figure 8:**
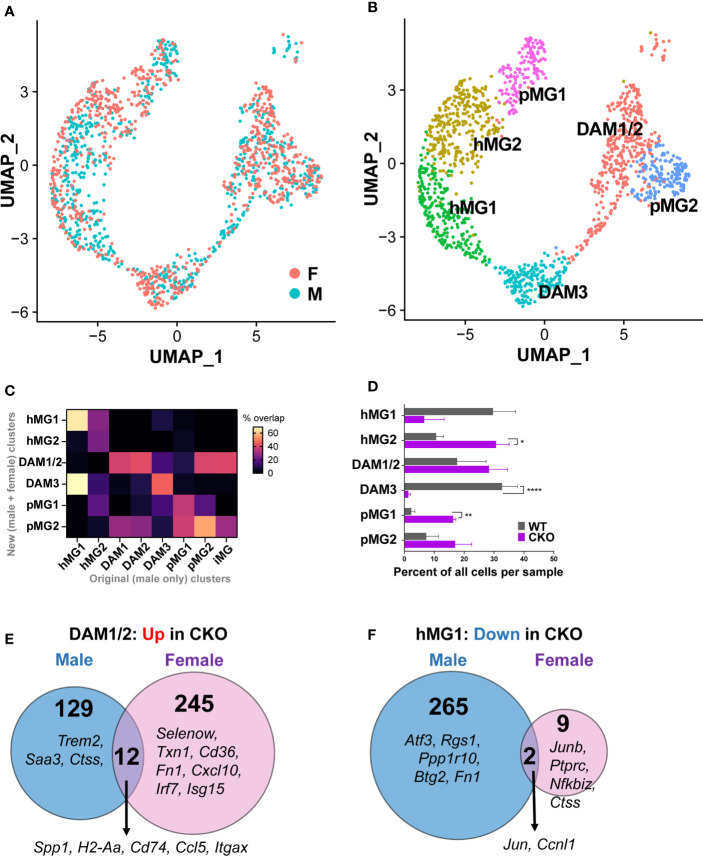
Unique and overlapping roles of p38α in female vs. male microglia. Microglia were isolated from female p38αCKO*^Cx3cr1-ER^* (CKO; n=3) and WT control mice (n=3) during peak EAE and analyzed by scRNAseq as described in the Materials and Methods. scRNAseq data from male CKO (n=3) and WT microglia (n=3) (**-**) were integrated and reanalyzed together with the female data using Seurat v3. UMAP-based two-dimensional projection of single cells, shown by sex **(A)** or by cluster identity **(B)**. **(C)** Comparison of cluster identities originally defined in for male microglia (X axis), with newly defined clusters of male and female microglia (Y axis). The heatmap indicates percent overlap between the cluster marker genes for each cluster from each analysis, normalized to the maximum possible percent overlap given the size of each gene set. **(D)** The distribution of female WT and CKO cells across each cell cluster, expressed as the relative percent of each cell cluster, determined by dividing the number of cells in a given cluster by the total number of cells in a biological replicate. Significance of differences between WT and CKO are indicated as follows: *P < 0.05; **P < 0.01; ****P < 0.0001. **(E)** Comparison of DEGs upregulated (P<0.05, log2FC>0.2) in p38αCKO relative to WT in male vs. females, in microglial cluster DAM1/2. **(F)** Comparison of DEGs downregulated (P<0.05, log2FC < -0.2) in p38αCKO relative to WT in male vs. females, in microglial cluster hMG1.

Our initial single cell transcriptomic analyses were focused on microglia isolated from male mice, where p38α deficiency exacerbated EAE. Given the divergent impact of microglial p38α deficiency on EAE severity between males and females (**Figure 3**), we next performed scRNAseq on microglia isolated from female p38αCKO*^Cx3cr1-ER^* mice and WT controls (p38α^f/f^ Cre- littermates) at peak EAE using FACS, followed by single cell RNA sequencing. To perform direct comparisons, we combined and reanalyzed the male and female data together and performed an anchored clustering analysis in Seurat3 (see *Materials and Methods*), excluding the T cell and neutrophil clusters to focus on microglia. This analysis revealed a comparable overall distribution of male and female cells across clusters (**Figure 8A**), with 6 total microglial clusters (**Figure 8B**). In order to maintain consistency with our previous analysis in males, we calculated overlap for cluster marker genes for males (**Supplementary File 2**) and the newly defined cluster marker genes for the male-female combined analysis (**Supplementary File 4**). There was a high degree of similarity for cluster identities that allowed us to designate clusters as previously defined (hMG1, hMG2, pMG1, pMG2, and DAM3), and the previous DAM1, DAM2, and iMG clusters were grouped into a single cluster (termed DAM1/2) in the grouped male-female analysis (**Figure 8C**). This analysis confirmed the transitional nature of DAM3, which exhibited a significant overlap with hMG1 (**Figure 8C**), and grouped between hMG and DAM on the UMAP plot (**Figure 8B**). It also confirmed that the pMG2 cluster represents a proliferative subpopulation of DAM (**Figures 8B, C**). Taken together, these results demonstrate that microglia in the inflamed CNS of male and female mice exist on an overall similar spectrum of transcriptional states, ranging from homeostatic microglia to DAM.

We next asked whether p38α deficiency in female microglia exhibited distinct effects compared with the effects in males. In terms of cell distribution across cell clusters, we found that p38α deficiency in females resulted in the depletion the DAM3 cluster, with a corresponding increase in the homeostatic hMG2 and pMG1 clusters, and no major effect on the main DAM1/2 cluster (**Figure 8D**), unlike what was seen in males (**Figure 7B**). To determine whether p38α deficiency exerted sex-specific effects on microglial gene expression, we performed a p38αCKO *vs*. WT DEG analysis across the six microglial clusters, separately in females and in males (see Materials and Methods). This analysis revealed that p38α deficiency resulted in highly divergent cluster-specific DEGs identified in females *vs*. males (**Supplementary Files 5, 6**). For example, in the key DAM1/2 cluster, with regard to genes upregulated in p38αCKO, only 12 DEGs were common to both male and females, including antigen presentation-associated and DAM-associated *Spp1, H2-Aa*, *H2-Eb1, Cd74*, *Ccl5*, and *Itgax*. Male and female p38αCKO DAM1/2 microglia exhibited a distinct repertoire of upregulated genes (141 and 257 genes respectively). For males this included the DAM-associated genes *Trem2, Saa3, and Ctss* in males, and MHC class I genes (*H2-Q4, H2-Q6*) (**Figure 8E**). In females, this included tissue protective genes such as *Txn1* (thioredoxin 1), *Fn1* (fibronectin 1), *Cd36*, and *Selenow* (selenoprotein W), as well as interferon responsive genes *Irf7*, *Isg15*, *Ifi211*, *Cxcl10*, and others (**Figure 8F**). In the hMG1 cluster, many more genes were downregulated in p38αCKO males compared with females (267 *vs*. 11), including the homeostatic genes *Atf3*, *Rgs1*, *Ppp1r10*, and *Btg2* (identified above) (**Figure 8E**). Similarly distinct transcriptional responses to p38α deficiency were seen between males and females across other clusters (**Supplementary Files 5, 6**). Taken together, these results demonstrate that p38α deficiency in microglia exerts distinct transcriptional effects in females compared with males, which are in line with the more pronounced exacerbating impact of microglial p38α deficiency on EAE severity in males.

## Discussion

Sex differences in MS and EAE are well-documented, and are thought to be driven by a combination of effects of sex hormones and sex chromosomes (52, 53). Beyond the action of these classic sex-specific mediators, surprisingly little is known about other sex-specific molecular pathways. Here, we uncovered a sex-specific signaling pathway in microglia that is protective in EAE, in contrast to its disease-promoting role in peripheral myeloid cells. We originally identified a potential sex difference in the role of p38α in EAE when we demonstrated that deletion of this kinase broadly in the myeloid lineage using LysM-Cre (likely including microglia) was protective in females but not males (24). We now extended these findings to determine that p38α in peripheral myeloid cells promotes EAE in both males and females, but that the previously observed sex difference is actually driven by an opposing protective effect of p38α in microglia in males. Thus, when both microglia and peripheral myeloid cells are targeted using LysM-Cre or constitutive *Cx3cr1*-Cre, we surmise that these opposing effects in males cancel each other out (see model in **Table 2**).

**Table 2:** A model for cell specific effects of p38α deletion in microglia and/or peripheral myeloid cells.

p38α-deficient cell type	Females	Males
Peripheral myeloid cells (Cx3cr1-Cre BM→ B6 host chimera), **Figure 2**	↓ EAE	↓ EAE
Microglia (**Figure 3**) (Cx3cr1-ER-Cre)	=EAE	↑ EAE
Peripheral myeloid cells & microglia (Cx3cr1-Cre or LysM-Cre) (**Figure 1** and published)	↓ EAE	=EAE

↓, ↑, and "=" indicate decreased, increased, or unchanged EAE severity, respectively.

We previously demonstrated using gonadectomy experiments that the original myeloid cell p38α-mediated sex difference in EAE was dependent on the role of sex hormones, presumably estrogens and androgens (24). We recently demonstrated that this difference was driven by signaling through estrogen receptor alpha in myeloid cells, but unexpectedly in males and not females (30), most likely *via* conversion of androgens to estrogens and subsequent estrogen receptor activation. Our findings in the present study, demonstrating that the p38α-mediated sex difference is in fact driven by a difference in male microglia, are completely compatible, and further highlight the known role for microglia in sensing *endogenous* estrogens that may be locally produced in the CNS (54). Moreover, our upstream regulator analysis of p38α-dependent DEGs in male microglial clusters identified ESR1 as a potential positive regulator (**Figure S7B**). Effects of *exogenous* estrogens on microglia have been previously shown in the EAE model (55–57), although it has not yet been demonstrated whether these are direct or indirect effects. Future studies will need to address the role of estrogen receptors or the androgen receptor in driving the sex-specific role of p38α in microglia.

Our single cell transcriptional profiling experiments in p38αCKO*^Cx3cr1-ER^* mice in this study were initially performed in male mice, in which microglial-specific p38α deletion showed a significant effect in EAE. Our subsequent bulk and single transcriptomic analysis in females determined that p38α regulates distinct sets of genes in male *vs*. female microglia. The loss of p38α promoted predominantly proinflammatory pathways in males, but not females (**Figure S3** and **Supplementary File 1**). Interestingly, we did find a conserved group of genes that were p38α-dependent in both sexes, including *Ppp1r10, Adamts1, Hmox1*, and several histone cluster genes, all of which were also identified by scRNAseq (**Figure S6H**). As discussed below, it is possible that these are regulatory genes that maintain the homeostatic/resting transcriptional program in microglia, but their loss in female, but not male, p38α-deficient microglia can be compensated by other pathways. Notably, previous studies have identified a p38α-dependent pathway in microglia that mediated pain hypersensitivity in male but not female mice (58), suggesting that differential “wiring” of this signaling pathway in male and female microglia may be a general feature of this cell type. In further support of this notion, several studies have recently documented profound transcriptional differences between male and female adult microglia in the steady state (59–62), and sex-specific effects of microglia perturbation on EAE severity have also been reported (63).

While p38α was originally identified as a pro-inflammatory signaling molecule in myeloid cells, more recent studies have provided compelling evidence for an anti-inflammatory role (21), which could be consistent with our current findings that deletion of p38α in microglia exacerbates EAE. We and others have demonstrated that in macrophages the anti-inflammatory functions of this kinase are largely driven by p38α-dependent production of the anti-inflammatory cytokine IL-10 and/or by induction of the MAPK phosphatase DUSP-1 (64, 65). In the present study, we did not find a difference in expression of *Il10* (encoding IL-10), and in fact this gene was expressed at minimal levels in microglia as determined by scRNAseq and bulk transcriptomics (data not shown). *Dusp1* (encoding DUSP-1) in females was highly and significantly downregulated (**Supplementary File S5**), and in males showed a modest trend (P=0.067) towards decreased expression in the hMG1 cluster (data not shown). DUSP-1 was also identified indirectly as a predicted upregulated node in p38α-deficient microglia (**Figure S7B**), and *Dusp1* represents a marker of homeostatic microglia (**Supplementary File 2**). Notably, a different phosphatase, *Ppp1r10*, was also prominently downregulated in male and female p38α-deficient microglia (**Figure S6H** and **Figure S3A**), suggesting that, similar to macrophages, upregulation of phosphatase expression may be a general negative feedback mechanism for this pathway in microglia. Another known target of p38α is tristetraprolin (TTP), encoded by the *Zfp36* gene, which is regulated by p38α at the transcriptional and post-transcriptional levels (21). This gene is downregulated in p38α-deficient microglia (DAM3 cluster) in males (**Figure S6H**), and also represents a marker of homeostatic microglia (**Supplementary File 2**). As discussed above, several other p38α-dependent genes in microglia may represent key determinants of its regulatory function, including *Atf3*, *Btg2*, *Rgs1*, *Nfkbiz*, as well TGFβ-related genes *Tgfbi, Tgif*. Taken together, our results suggest that specific regulatory/anti-inflammatory targets of p38α in microglia differ substantially from that in macrophages, but the general features of feedback mechanisms may be conserved. Future studies will identify which of the genes differentially expressed in p38α-deficient microglia are functionally responsible for the EAE phenotype observed.

The p38 MAPK signaling pathway represents a readily druggable therapeutic target, and much progress has been made in developing pharmacologic inhibitors to this end (21). Previous studies, including our own, have also demonstrated a non-sex specific EAE-promoting role of p38α signaling in T cells (66, 67), dendritic cells (24, 68), and neuroectoderm-lineage CNS cells (69). Moreover, inhibition of p38α in the oligodendrocyte lineage promoted remyelination in the cuprizone intoxication model (70). However, our present findings demonstrate sex- and cell type-specific opposing roles for p38α, which highlight the complexity of targeting this pathway therapeutically in MS or other autoimmune diseases. Our results also accentuate the critical need to stratify and report clinical trial data by sex, so that sex-specific effects may be more readily discerned. Finally, our results suggest that therapeutic efficacy of various treatments can be improved by cell type- and sex-specific targeting.

We used several different approaches to investigate the role of p38α in peripheral myeloid cells *vs*. microglia. While transplantation of bone marrow from p38αCKO*^Cx3cr1-Cre^* mice to WT mice successfully limited the Cre activity to peripheral myeloid cells, the reverse bone marrow chimera was problematic in that we observed ectopic Cre activity in CD45-negative cells and loss of reporter activity in microglia. The former is consistent with a recent report of ectopic expression of *Cx3cr1-Cre* in multiple other CNS cell types (likely other glia and/or neurons) (38), but the reasons for the latter are unclear, but may have to do with effects of irradiation on the p38αCKO*^Cx3cr1-Cre^* host. In this regard, the irradiation chimera approach has some additional limitations, since some microglial replacement by bone marrow-derived cells can occur in some settings (71).

In addition to illuminating the role of p38α in microglia in EAE, our findings using single cell transcriptomics contribute more broadly to the understanding of microglial heterogeneity during CNS autoimmunity. A recent study by Prinz and colleagues performed scRNAseq of microglia during EAE, identifying two homeostatic clusters (hMG1 and hMG2) and four disease-associated clusters (daMG1-4) (45), which is highly reminiscent of our results. Although a full list of signature genes for these clusters was not reported in this study, several top marker genes for daMG2-4 (but not hMG1, hMG2, and daMG1) were reported, which allow a qualitative comparison with our DAM clusters. In the Prinz study, daMG2 expressed high levels of *Cd74*, *B2m*, *Apoe*, *Cst7*, and *Ctsb*, which is consistent with a classic DAM phenotype, and highly similar to our DAM1 and DAM2 clusters (**Table 1** and **Supplementary File 2**). The daMG3 cluster expressed higher levels of *Cxcl10*, similar to pMG1 in our study, although it also overexpressed *Ccl4* and *Tnf*, which were markers for our “transitional” DAM3 cluster. The daMG4 cluster was marked by signature genes that overlapped with several different clusters in our analysis, *Itm2b* (in our iMG cluster), *Ctss* (in our DAM1 cluster), *Ccl5* (in our DAM2 cluster) and *Naaa* (in our DAM3) cluster. Thus, although the clusters do not match one-to-one, there is overall very close concordance between transition from homeostatic to DAM states, and it is possible that the specific clusters may be dependent on the software used for the clustering analysis, rather than representing absolute identities. With regard to mapping transition from homeostatic to inflammatory states, our study is the first (to our knowledge) to perform single cell trajectory analysis in the setting of EAE, although some trajectory is implied in the results of Prinz and colleagues. Here our results are also concordant at the broader level, with the progressive downregulation of homeostatic genes like *P2ry12* and *Tmem119*, and upregulation of DAM-associated genes like *Apoe* and *Cd74*. However, our results also reveal finer details of this transition, revealing not only inflammatory genes, but also adaptive changes (e.g. anti-oxidant, iron homeostasis, and ECM-remodeling genes) in the DAM, and further suggesting that distinct homeostatic states also preferentially transition to specific, although somewhat convergent DAM states. Future studies will be necessary to test this intriguing possibility experimentally. Importantly, our results are also highly concordant with recent human studies that performed single-nucleus RNAseq of PPMS lesions, identifying unique MS-associated microglial signatures, which not only exhibited elevated classic DAM genes like *APOE*, but also distinct microglial clusters associated with either iron homeostasis or elevated lipid phagocytosis (72).

## Data Availability Statement

The datasets presented in this study can be found in online repositories. The names of the repository/repositories and accession number(s) can be found below: NCBI GEO GSE180864 (microarray) and GSE185045 (scRNAseq).

## Ethics Statement

The animal study was reviewed and approved by IACUC at the University of Vermont.

## Author Contributions

Experimental design, DK and MM. Execution of experiments, MM, DK, KL, TM, SC, and SV. Data analysis and figure preparation, MM, AR, JB, SF, and DK. Manuscript writing and editing, MM, AR, SF, JB, and DK. Securing funding, SF and DK. All authors contributed to the article and approved the submitted version.

## Funding

The research reported here was supported by research grant RR-1602-07780 from the National Multiple Sclerosis Society and a technology development initiative pilot project (under grant U54 GM115516 from the National Institutes of Health for the Northern New England Clinical and Translational Research network) to DK. Research performed at the Flow Cytometry and Cell Sorting Facility at UVM was partially supported by NIGMS P20GM103496.

## Conflict of Interest

The authors declare that the research was conducted in the absence of any commercial or financial relationships that could be construed as a potential conflict of interest.

## Publisher’s Note

All claims expressed in this article are solely those of the authors and do not necessarily represent those of their affiliated organizations, or those of the publisher, the editors and the reviewers. Any product that may be evaluated in this article, or claim that may be made by its manufacturer, is not guaranteed or endorsed by the publisher.
