# Supplementary material for: p38 MAP Kinase Signaling in Microglia Plays a Sex-Specific Protective Role in CNS Autoimmunity and Regulates Microglial Transcriptional States
Source: Front Immunol. 2021 Oct 11;12:715311. doi: 10.3389/fimmu.2021.715311 (PMC8542909; doi:10.3389/fimmu.2021.715311)
Supplement: Supplementary file 1 [file DataSheet_1.pdf]

INDEX OF SUPPLEMENTARY MATERIAL (Datasheet 1)

Supplementary Figures:

*All supplementary figures are provided as a single PDF file (**Datasheet 8** - Supplementary Figures), with legends included.*

Figure S1. Schematic of mouse models used in the study.

Figure S2. Bone marrow chimeric WT→p38αCKO^{Cx3cr1} mice show low specificity of targeting to microglia, variable EAE, and an apparent lack of effect of p38α deletion.

Figure S3. p38α deficiency differentially impacts male vs. female microglia.

Figure S4. Single cell transcriptional analysis of CNS microglia in EAE.

Figure S5. Additional transcriptional trajectory analysis of microglial states in males.

Figure S6. Identification of p38α-regulated gene signatures in male microglia using scRNAseq.

Figure S7. Pathway analysis of p38α-regulated gene expression across multiple microglial clusters.

Supplementary Files (Excel format):

Supplementary File 1. (**Datasheet 2**). DEGs between p38α and WT bulk microglia identified by microarray, in males and females. Individual tabs show male DEGs, female DEGs, and overlap, respectively.

Supplementary File 2. (**Datasheet 3**).Cluster markers identified by scRNAseq in male microglia.

Supplementary File 3. (**Datasheet 4**).Trajectory analysis of male microglia using scRNAseq. Individual tabs contain analysis for individual branches of the trajectories, as indicated.

Supplementary File 4. (**Datasheet 5**).Cluster markers identified by scRNAseq for male and female combined analysis of microglia.

Supplementary File 5. (**Datasheet 6**).Cluster-specific DEGs identified by scRNAseq in p38α-deficient male microglia. Individual tabs show DEGs for each cluster, as indicated.

Supplementary File 6. (**Datasheet 7**).Cluster-specific DEGs identified by scRNAseq in p38α-deficient female microglia. The first tab shows overlapping and unique between male and female DEGs by cluster. Remaining individual tabs show female DEGs for each cluster, as indicated.
