# Supplementary material for: p38 MAP Kinase Signaling in Microglia Plays a Sex-Specific Protective Role in CNS Autoimmunity and Regulates Microglial Transcriptional States
Source: Front Immunol. 2021 Oct 11;12:715311. doi: 10.3389/fimmu.2021.715311 (PMC8542909; doi:10.3389/fimmu.2021.715311)
Supplement: Supplementary file 8 [file DataSheet_8.pdf]

Supplementary Figures

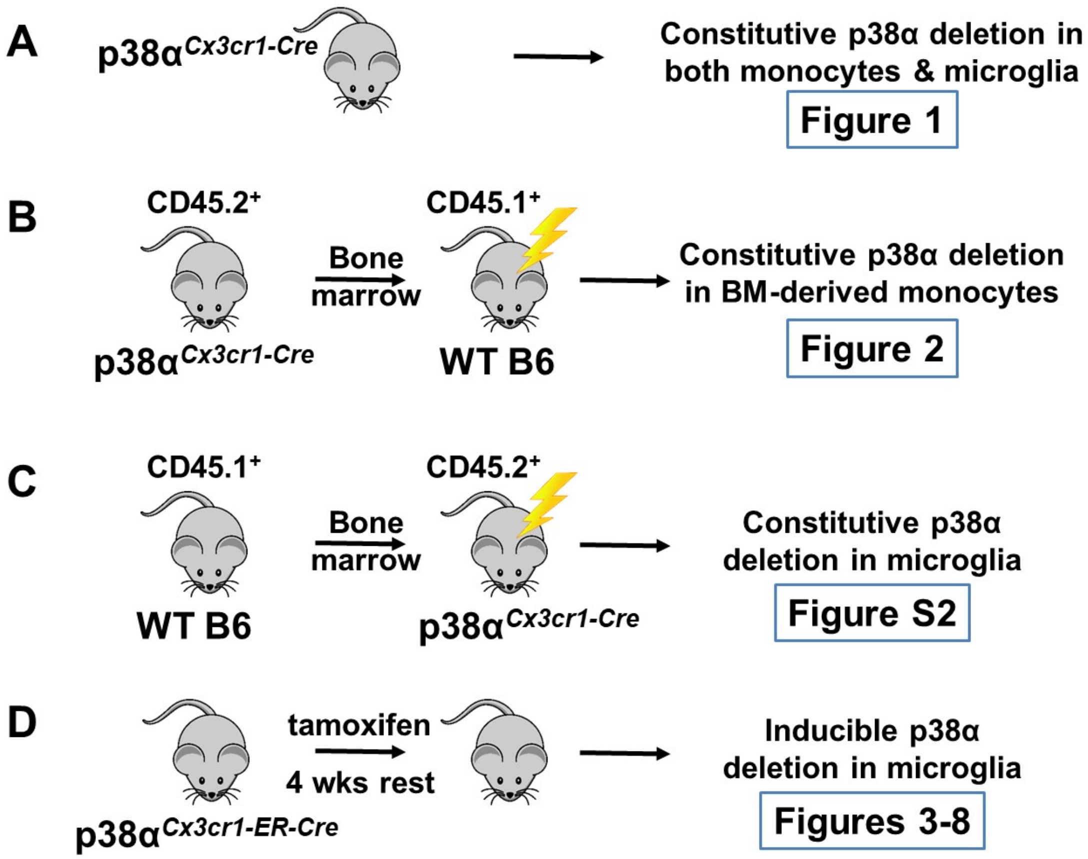

Figure S1. Schematic of mouse models used in the study.

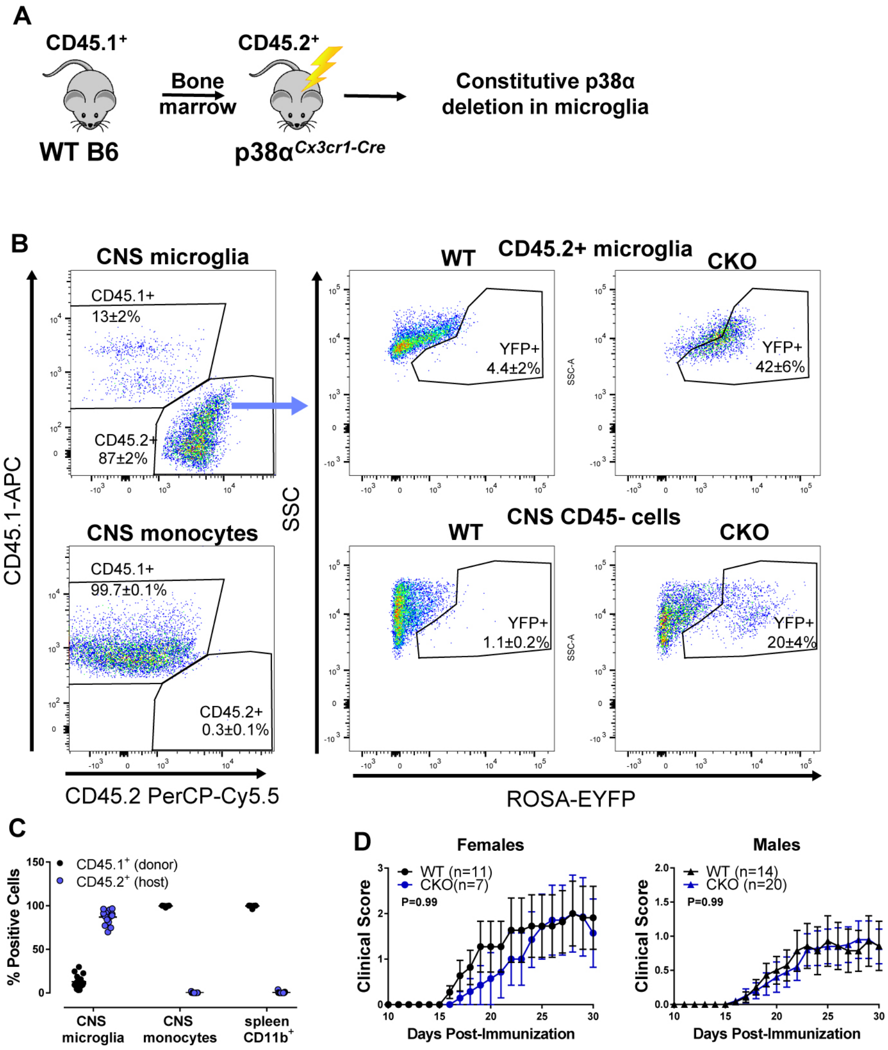

Figure S2. Bone marrow chimeric WT→p38→CKO^{Cx3cr1} mice show low specificity of targeting to microglia, variable EAE, and an apparent lack of effect of p38→deletion. Bone marrow chimeric mice were constructed by the transplantation of WT CD45.1^{+} bone marrow to irradiated CD45.2^{+} p38αCKO^{Cx3cr1} or WT littermate Cre-negative recipients (see Materials and Methods). (**A**) CNS cells were isolated from chimeric WT→p38αCKO^{Cx3cr1} BM (CKO) and WT BM→WT (WT) male mice at day 30 post EAE induction and analyzed by flow cytometry. Left side - representative plots showing CD45.1 (donor) and CD45.2 (host) marker expression for CNS microglia (CD45^{+} CD11b^{+} CX3CR1^{+} Ly6C^{-} Ly6G^{-} MHCII^{-} CD11c^{-}) and monocytes (CD45^{+} CD11b^{+} Ly6C^{+}). Right side – representative plot showing EYFP reporter expression in CD45.2^{+} microglia (top; gated as above) and in CNS CD45-negative cells (bottom) in WT and CKO mice. Frequencies are shown as mean ± SEM. (**B**) Quantification of frequencies of CD45.1^{+} (host) and CD45.2^{+} (donor) cells in CNS monocytes and microglia (gated as in (**A**)) and spleen CD45^{+} CD11b^{+} myeloid cells. (**C**) EAE course in chimeric WT BM→p38αCKO^{Cx3cr1} BM (CKO) and WT BM→WT (WT) male and female mice. P value indicates significance of difference in EAE course between WT and CKO, calculated as calculated as in **Fig. 1.** Sample size for each sex/genotype is indicated in parentheses in the panel legends.

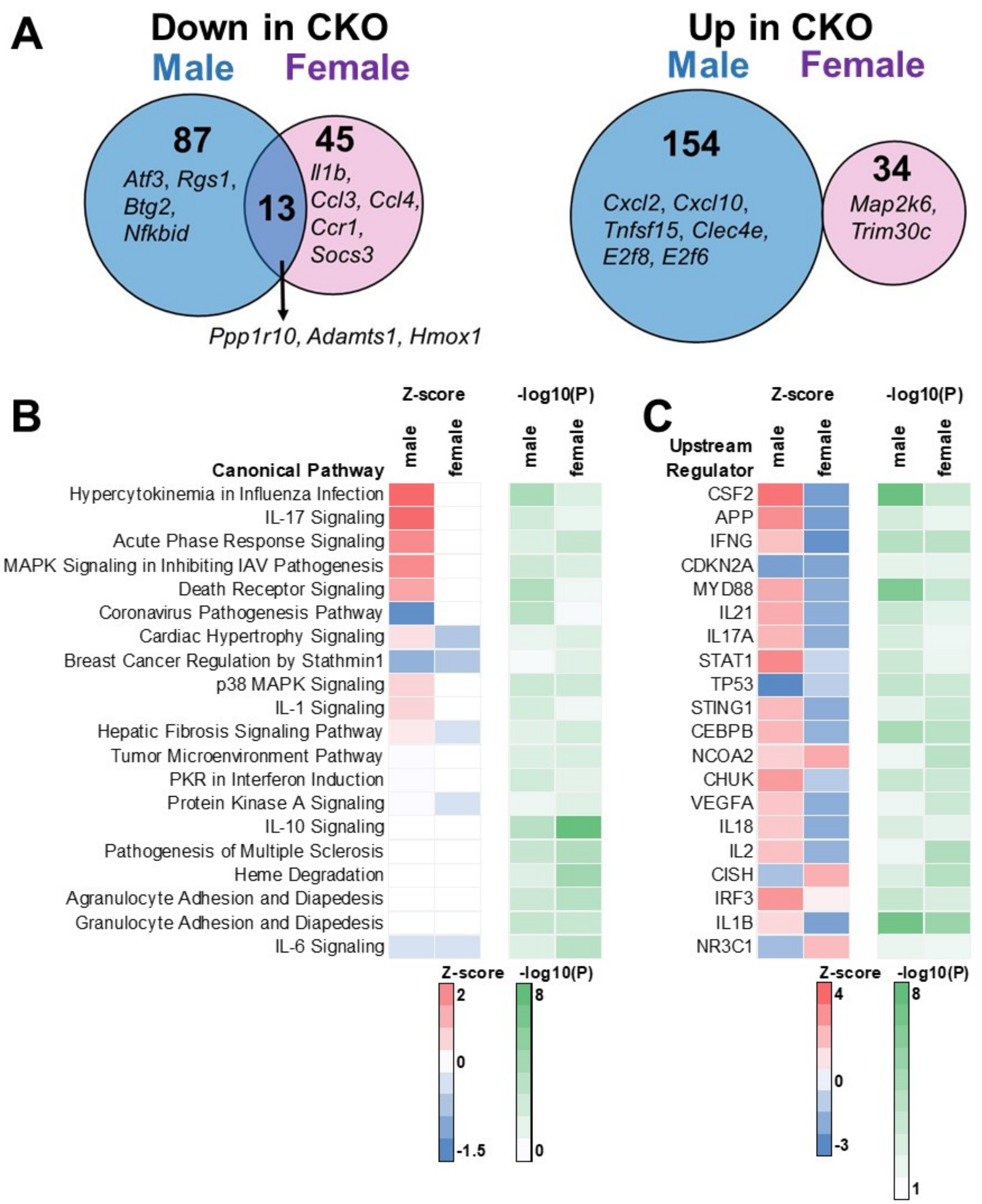

Figure S3. p38α deficiency differentially impacts male vs. female microglia. Female and male p38αCKO$^{Cx3cr1\text{-}ER}$ (CKO; n=5,4) and WT control (n=5,6) mice were treated with tamoxifen for 4 days, followed by a 4 week rest period, at which point EAE was induced. On day 21 post-EAE induction, microglia were isolated and analyzed by microarray as described in **Fig. 5**. (**A**) Up and down-regulated genes in CKO vs. WT microglia passing the threshold of |FoldChange|>2 and P<0.05 for each sex are shown. Select genes are annotated. (**A** and **B**) Genes differentially expressed in CKO vs. WT in each sex were subjected to bioinformatic analysis using Ingenuity software (see Materials and Methods). Top 20 canonical pathways (**B**) and upstream regulators (**C**), as identified by sum of |Z scores|, with positive Z-score indicating enhanced predicted activity in CKO.

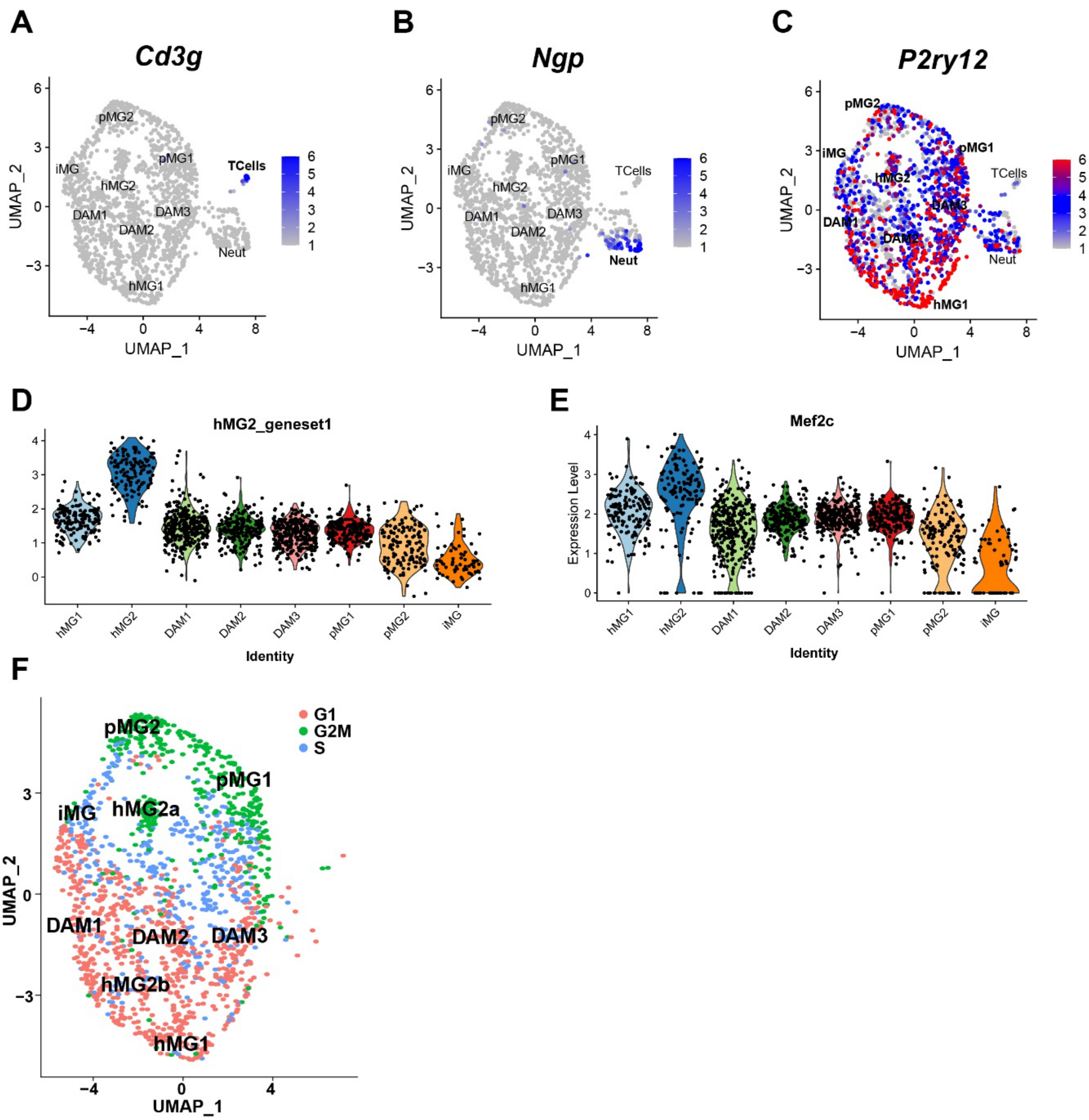

Figure S4. Single cell transcriptional analysis of CNS microglia in EAE. Male microglia were isolated and analyzed by scRNAseq as described in **Fig. 6**. (**A-C**) Feature plots demonstrating expression of select indicated genes across the Seurat v3-defined cell clusters. (**D**) Relative expression of the hMG2 signature gene module (defined in **Fig. 6B**) across microglial cell clusters. (**E**) Relative expression of *Mef2c* across microglial cell modules. (**F**) Seurat v3-based cell cycle analysis of the 8 microglial clusters defined in **Fig. 6A**.

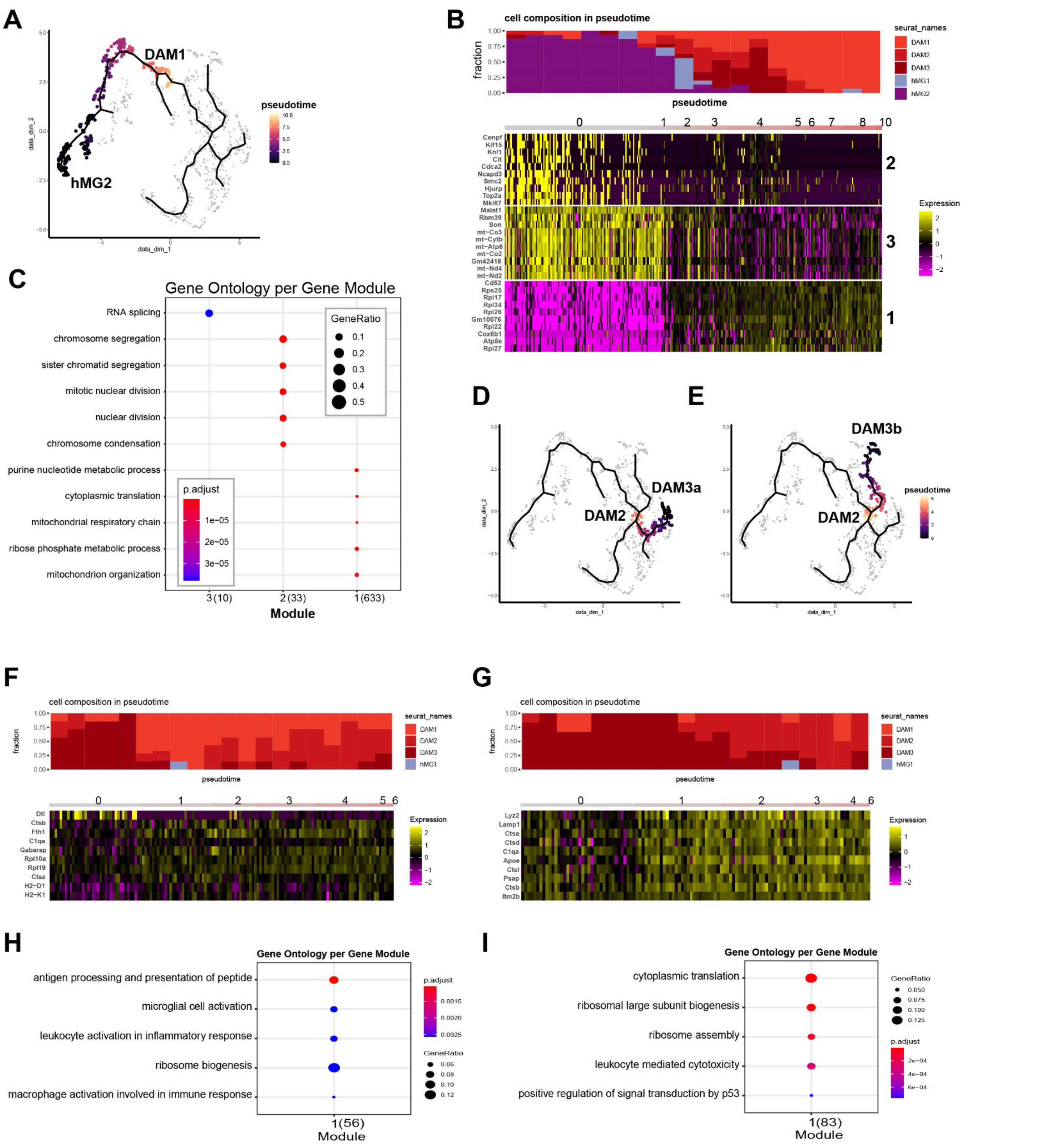

Figure S5. Additional transcriptional trajectory analysis of microglial states in males. Seurat v3-defined microglial clusters were analyzed by Monocle 3 as described in **Fig. 7**. Three distinct trajectories and their associated gene modules are shown: hMG2 > DAM1 (**A**-**C**), DAM3a > DAM2 (**D**,**F**, and **H**), and DAM3b > DAM2 (**E**, **G**, and **I**).

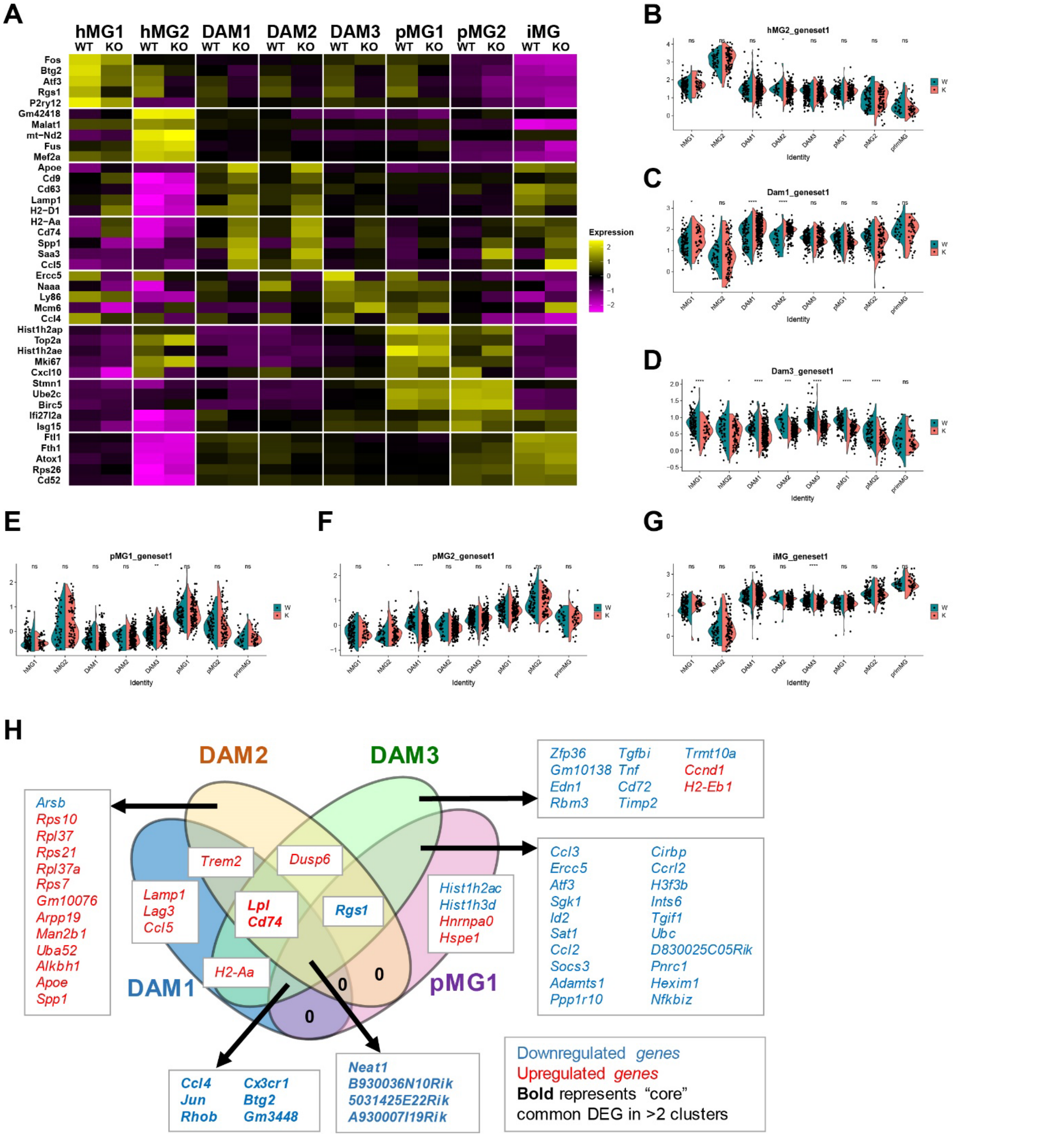

Figure S6. Identification of p38α-regulated gene signatures in male microglia using scRNAseq. ScRNAseq analysis of male microglia was performed as described in Materials and Methods, using Seurat v3 software. (**A**) Heatmap of signature gene markers for 8 microglial cell clusters identified in **Fig. 6B**, shown by genotype (WT vs. CKO). Row normalized gene expression is shown. Average gene expression of all cells in each cluster is shown. (**B-G**) Violin plots demonstrating the expression of the indicated cluster module genes (as defined in **Fig. 6B**), across 8 microglial clusters. (**H**) Venn diagram illustrating overlap between genes differentially expressed in CKO vs. WT cells across 4 microglial cell clusters (DAM1-3, and pMG1).

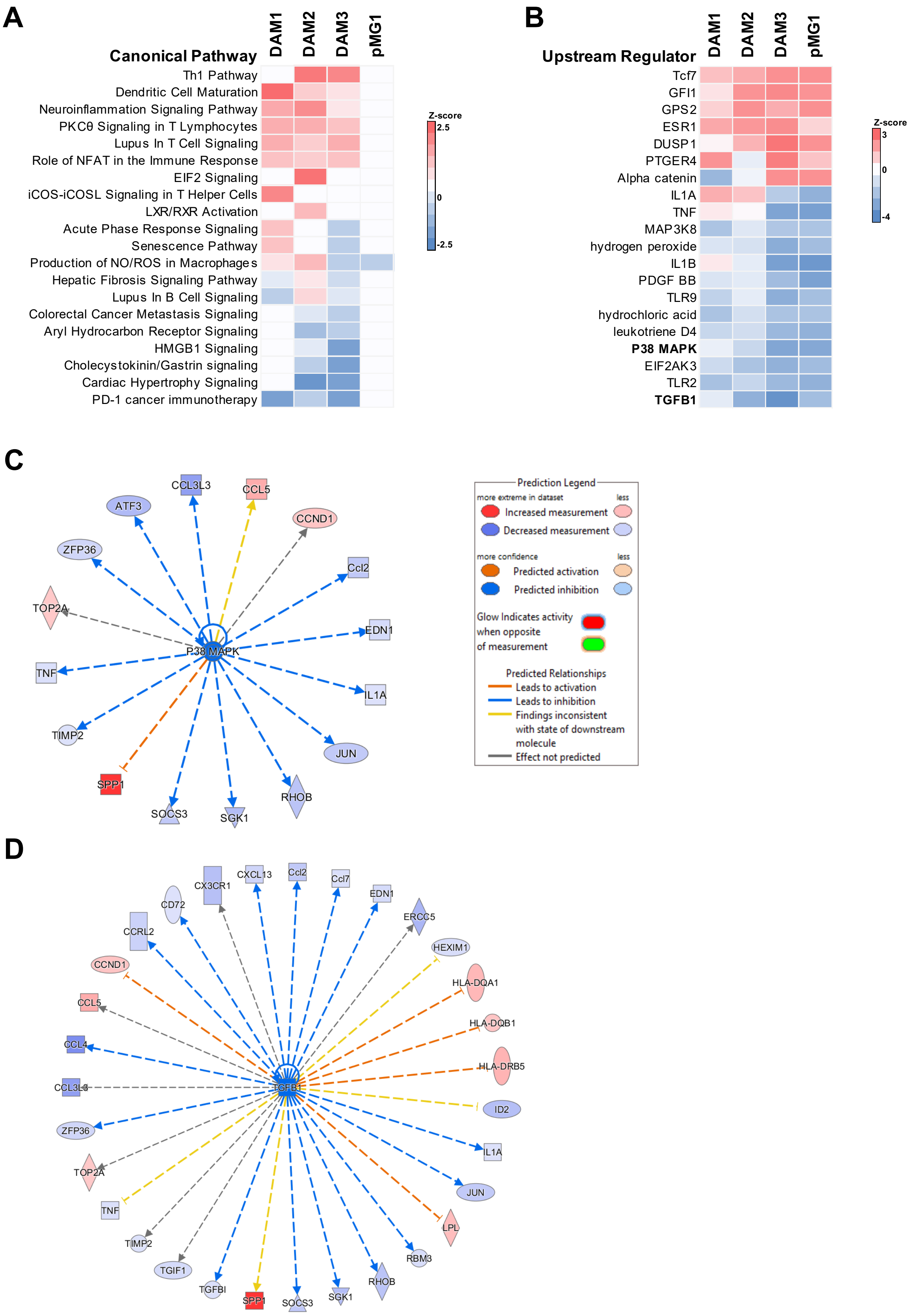
A
Canonical Pathway
DAM1
DAM2
DAM3
pMG1
Th1 Pathway
Dendritic Cell Maturation
Neuroinflammation Signaling Pathway
PKCθ Signaling in T Lymphocytes
Lupus In T Cell Signaling
Role of NFAT in the Immune Response
EIF2 Signaling
iCOS-iCOSL Signaling in T Helper Cells
LXR/RXR Activation
Acute Phase Response Signaling
Senescence Pathway
Production of NO/ROS in Macrophages
Hepatic Fibrosis Signaling Pathway
Lupus In B Cell Signaling
Colorectal Cancer Metastasis Signaling
Aryl Hydrocarbon Receptor Signaling
HMGB1 Signaling
Cholecystokinin/Gastrin signaling
Cardiac Hypertrophy Signaling
PD-1 cancer immunotherapy
Z-score
2.5
0
-2.5
B
Upstream Regulator
DAM1
DAM2
DAM3
pMG1
Tcf7
GFI1
GPS2
ESR1
DUSP1
PTGER4
Alpha catenin
IL1A
TNF
MAP3K8
hydrogen peroxide
IL1B
PDGF BB
TLR9
hydrochloric acid
leukotriene D4
P38 MAPK
EIF2AK3
TLR2
TGFB1
Z-score
3
0
-4
C
CCL3L3
CCL5
ATF3
CCND1
ZFP36
Ccl2
TOP2A
EDN1
P38 MAPK
TNF
IL1A
TIMP2
JUN
SPP1
SOCS3
SGK1
RHOB
Prediction Legend
more extreme in dataset
less
Increased measurement
Decreased measurement
more confidence
less
Predicted activation
Predicted inhibition
Glow Indicates activity when opposite of measurement
Predicted Relationships
Leads to activation
Leads to inhibition
Findings inconsistent with state of downstream molecule
Effect not predicted
D
CX3CR1
CXCL13
Ccl2
Ccl7
CD72
EDN1
ERCC5
CCRL2
HEXIM1
CCND1
HLA-DQA1
CCL5
HLA-DQB1
CCL4
HLA-DRB5
CCL3L3
TGFB1
ID2
ZFP36
IL1A
TOP2A
JUN
TNF
LPL
TIMP2
RBM3
TGIF1
RHOB
TGFBI
SPP1
SOCS3
SGK1

Figure S7. Pathway analysis of p38α-regulated gene expression across multiple microglial clusters. Genes differentially expressed between WT and p38α-deficient male microglia across four cell clusters (DAM1,2,3 and pMG1) were identified by scRNAseq as described in **Fig. 8**, followed by canonical pathway (**A**) and upstream regulator (**B**) analyses using Ingenuity software (see Materials and Methods). Shown are the top 20 pathways or regulators exhibiting the largest cumulative Z scores across the 4 cell clusters. Predicted regulation of DEGs in DAM3 of putative downstream genes belonging to the P38 MAPK pathway (**C**) or TGFB1 pathway (**D**). Differential expression is overlaid on the gene icons; arrows indicate predicted direction regulation and consistency with observations.
